# Systematic review and meta-analysis of the diagnostic accuracy of spontaneous nystagmus patterns in acute vestibular syndrome

**DOI:** 10.3389/fneur.2023.1208902

**Published:** 2023-06-16

**Authors:** Martina Wüthrich, Zheyu Wang, Carlos Mario Martinez, Sergio Carmona, Georgios Mantokoudis, Alexander Andrea Tarnutzer

**Affiliations:** ^1^Faculty of Medicine, University of Zurich, Zurich, Switzerland; ^2^Division of Quantitative Sciences, Sidney Kimmel Comprehensive Cancer Center, Johns Hopkins University School of Medicine, Baltimore, MD, United States; ^3^Department of Biostatistics, Johns Hopkins Bloomberg School of Public Health, Baltimore, MD, United States; ^4^Hospital Jose Maria Cullen, Santa Fe, Argentina; ^5^Fundación San Lucas para la Neurosciencia, Rosario, Argentina; ^6^Department of Otorhinolaryngology, Head and Neck Surgery, Inselspital, Bern University Hospital, University of Bern, Bern, Switzerland; ^7^Department of Neurology, Cantonal Hospital of Baden, Baden, Switzerland

**Keywords:** bedside diagnostic accuracy, nystagmus, stroke, vestibular, vertigo, dizziness

## Abstract

**Objectives:**

For the assessment of patients presenting with acute prolonged vertigo meeting diagnostic criteria for acute vestibular syndrome (AVS), bedside oculomotor examinations are essential to distinguish peripheral from central causes. Here we assessed patterns of spontaneous nystagmus (SN) observed in AVS and its diagnostic accuracy at the bedside.

**Methods:**

MEDLINE and Embase were searched for studies (1980–2022) reporting on the bedside diagnostic accuracy of SN-patterns in AVS patients. Two independent reviewers determined inclusion. We identified 4,186 unique citations, examined 219 full manuscripts, and analyzed 39 studies. Studies were rated on risk of bias (QUADAS-2). Diagnostic data were extracted and SN beating-direction patterns were correlated with lesion locations and lateralization.

**Results:**

Included studies reported on 1,599 patients, with ischemic strokes (*n* = 747) and acute unilateral vestibulopathy (*n* = 743) being most frequent. While a horizontal or horizontal-torsional SN was significantly more often found in peripheral AVS (pAVS) than in central AVS (cAVS) patients (672/709 [94.8%] vs. 294/677 [43.4%], *p* < 0.001), torsional and/or vertical SN-patterns were more prevalent in cAVS than in pAVS (15.1 vs. 2.6%, *p* < 0.001). For an (isolated) vertical/vertical-torsional SN or an isolated torsional SN specificity (97.7% [95% CI = 95.1–100.0%]) for a central origin etiology was high, whereas sensitivity (19.1% [10.5–27.7%]) was low. Absence of any horizontal SN was more frequently observed in cAVS than in pAVS (55.2 vs. 7.0%, *p* < 0.001). Ipsilesional and contralesional beating directions of horizontal SN in cAVS were found at similar frequency (28.0 vs. 21.7%, *p* = 0.052), whereas for pAVS a contralesional SN was significantly more frequent (95.2 vs. 2.5%, *p* < 0.001). For PICA strokes presenting with horizontal SN, beating direction was ipsilesional more often than contralesional (23.9 vs. 6.4%, *p* = 0.006), while the opposite was observed for AICA strokes (2.2 vs. 63.0%, *p* < 0.001).

**Conclusions:**

(Isolated) vertical and/or torsional SN is found in a minority (15.1%) of cAVS patients only. When present, it is highly predictive for a central cause. A combined torsional-downbeating SN-pattern may be observed in pAVS also in cases with isolated lesions of the inferior branch of the vestibular nerve. Furthermore, in cAVS patients the SN beating direction itself does not allow a prediction on the lesion side.

## 1. Introduction

Acute vertigo or dizziness is amongst the most frequent causes for primary care physician visits or emergency department (ED) consults, reflecting about 2.1–4.4% of all ED presentations (1–3). With its differential diagnosis being very broad and cutting across organ systems and specialties and ~4.4 million such visits to US EDs every year, resulting healthcare costs surpassed $10 billion (4). While in the majority of cases, underlying causes are benign and self-limited, at least 15% of all ED patients with these symptoms suffer from a dangerous underlying cause (2). The most common dangerous cause is stroke, accounting for ~3–5% of cases (5).

For patients presenting with acute prolonged vertigo or dizziness, meeting diagnostic criteria for acute vestibular syndrome (AVS), defined as a clinical syndrome of acute-onset, continuous vertigo, dizziness, or unsteadiness lasting days to weeks, and generally including features suggestive of new, ongoing vestibular system dysfunction [e.g., vomiting, nystagmus, severe postural instability; (6)], the combined use of targeted neuro-otologic bedside examination techniques such as HINTS (Head Impulse test, Nystagmus exam, Test of Skew) (7), HINTS+ (which adds a bedside test of hearing) (8), and assessing for gait/truncal instability (9) has been promoted. While HINTS(+) have excellent diagnostic accuracy for distinguishing benign, self-limited peripheral causes from dangerous, potentially life-threatening central causes (mostly strokes) when performed by appropriately trained clinicians, being superior than MRI-DWI in the first 24–48 h (10), its application by frontline health-care providers such as ED physicians or neurology residents lacking dedicated training may be not be feasible. This raises the question about the diagnostic accuracy of other bedside examination techniques that are more accessible to frontline healthcare providers such as assessing gait/truncal instability or describing spontaneous nystagmus (SN) patterns. While recent studies underline the value of grading gait/truncal instability in AVS patients (11, 12), the role of SN in acutely dizzy patients is less clear. Horizontal or horizontal-torsional SN beating away from the affected ear is a hallmark sign in patients with acute unilateral vestibulopathy (13) and may also be observed in patients with vertebrobasilar stroke (14). While it has been proposed that the SN pattern may be useful in the distinction between peripheral and central causes, with torsional and vertical SN pointing to latter one (15), the diagnostic accuracy of distinct SN patterns has not been systematically assessed.

Thus, the primary aim of this systematic review and meta-analysis was to report on the prevalence and diagnostic yield of SN patterns in acutely dizzy patients at the bedside. Furthermore, we also asked the question, to which extent the lesion location has an impact on the SN pattern and the beating direction (ipsilesional vs. contralesional) in lateralized lesions. We hypothesized that the presence of isolated torsional/torsional-vertical or vertical SN predicts a central cause and that the beating direction in cAVS patients critically depend on the location of the lesion.

## 2. Methods

### 2.1. Data sources and searches

We searched MEDLINE and Embase for English-language articles, using the following strategy and components: (1) vertigo/dizziness, (2) diagnostic accuracy of SN as assessed at the bedside, and (3) acute vestibular syndrome (including ischemic stroke, acute peripheral vestibulopathy). We also performed a manual search of reference lists from eligible articles and contacted corresponding authors where necessary. We did not seek to identify research abstracts from meeting proceedings or unpublished studies. We limited our search to articles published since 1980, when neuroimaging for stroke first became routine. Our search was updated through September 1st, 2022.

Being a systematic review of the literature and a meta-analysis, no ethical approval was necessary.

### 2.2. Study selection and quality assessment

Articles were selected by two independent screeners using pre-determined inclusion criteria and a structured process. Our focus was on studies examining the diagnostic accuracy of SN for distinguishing between peripheral and central causes of AVS in patient populations in the ED or on an acute inpatient ward in the acute stage. Not all studies provided specific numbers on delay from symptom onset to clinical examination of SN at the bedside and a subset of patients may have received delayed (i.e., after more than 72 h) examination only. In those studies providing single subject data, only those tested within 72 h were included. When aggregated numbers were provided only, we required testing within 72 h in at least 75% of patients. In those studies that did not provide specific numbers, patients presented with acute symptoms to the emergency department and bedside testing was performed as part of the initial assessment at the ED. Thus, it is reasonable to assume that these were patients with acute (i.e., < 72 h duration) symptoms.

We calculated interrater agreement on full-text inclusion using Cohen's kappa (16). We assessed the risk of bias and applicability concerns for all studies using QUADAS-2 criteria (17). The reference standard for “ruling out” stroke in a peripheral vestibular case was delayed (i.e., more than 48 h after symptom onset) magnetic resonance imaging with diffusion-weighted images (MRI-DWI); strokes could be “ruled in” using confirmatory neuroimaging, including computed tomography (CT) in the appropriate clinical context, but an unconfirmed clinical diagnosis was considered high risk of bias.

### 2.3. Data extraction, synthesis, and analysis

We report the diagnostic accuracy of various SN patterns observed at the bedside at primary (i.e., straight-ahead) gaze by visual inspection with fixation either preserved or removed, distinguishing horizontal/horizontal-torsional, vertical [upbeat nystagmus (UBN), downbeat nystagmus (DBN)] and torsional [clockwise (CW), counter-clockwise (CCW)] beating directions. Furthermore, we assess the beating direction of the horizontal component of the SN in relation to the side of the lesion (in case of lateralized lesions)—ipsilesional vs. contralesional—and evaluate the impact of the lesion location (e.g., in the vascular territory of the posterior inferior cerebellar artery [PICA] vs. the anterior inferior cerebellar artery [AICA]).

We calculated sensitivity, specificity, negative likelihood ratio (LR-) and positive likelihood ratio (LR+) for ED or acute-ward diagnoses for any central condition [including stroke, but not limited to this entity as the clinical presentation of other causes of cAVS is often overlapping (e.g., vestibular migraine)]. We present estimated proportions and, where appropriate, 95% confidence intervals (95% CI). Confidence intervals for sensitivities and specificities are calculated based on the Wilson method for binomial counts (18). For a study with zero cells, a continuity correction of 0.5 is added to all cells of that study. Confidence intervals for positive and negative likelihood ratios are calculated based on (19). A summary measure for each “finding” was calculated using a random effect model using the DerSimonian-Laird estimator (20, 21). Sensitivity of a study was calculated (and contributed to the random effect model) even when all other measures were not available (missing false-positives or true-negatives). Tests of heterogeneity were conducted based on Cochran's Q-test. Heterogeneity statistics (Cochran's *Q*-test) were calculated using R v4.2.1 (Foundation for statistical computing, Vienna, Austria) by a PhD biostatistician. For comparison of proportions, Fisher's exact test was used (Matlab R2022a, The MathWorks, USA) (22). This review is reported in accordance with PRISMA guidelines.

### 2.4. Data availability

Source data used for meta-analysis will be made available to others upon request to the corresponding author.

## 3. Results

**Figure 1:**
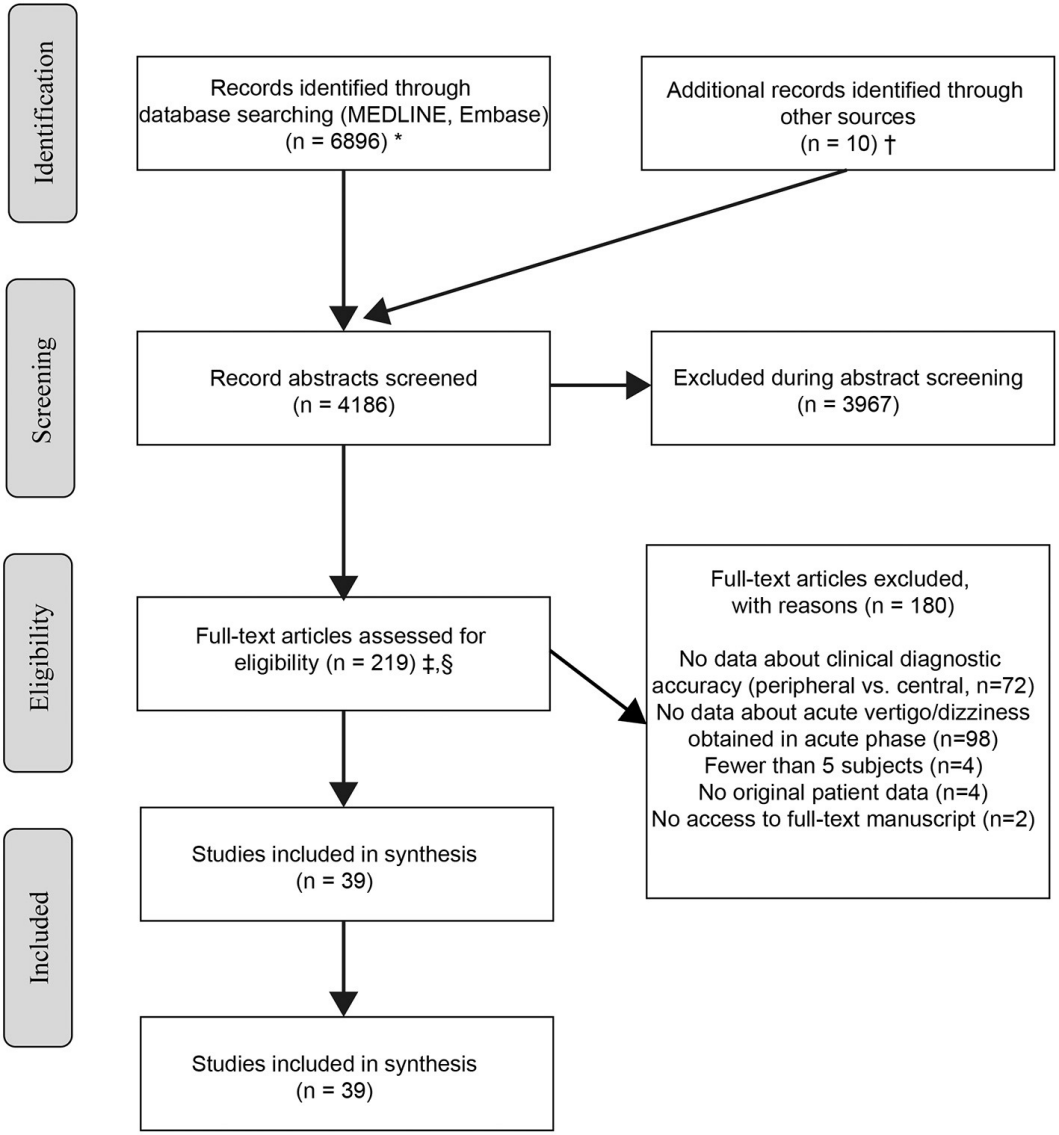
Citation search and selection flow diagram. ^*^MEDLINE was accessed via PubMed; Embase was accessed via embase.com. ^†^Hand search of citation lists from selected studies and investigator files identified 10 additional manuscripts for review. ^‡^Abstracts coded as “yes” or “maybe” by at least one reviewer were included in full-text review. ^§^After full-text evaluation by two reviewers, any differences were resolved by discussion and adjudication by a third, independent reviewer.

Our search identified 4,186 unique citations, of which 3,967 (94.8%) were excluded at the abstract level. Our independent raters had moderate initial agreement on inclusion of full-text manuscripts (kappa value 0.44). After resolving initial disagreements, 39/219 studies were considered eligible (Figure 1—PRISMA flow chart), representing 1.0% of the total. Among the 37 studies included in the final meta-analysis [two studies reporting preliminary data (7) or overlapping data (23) were excluded], the risk of bias and applicability concerns using the QUADAS-2 rating system was judged “high” or “unclear” in one (*n* = 10), two (*n* = 8), three (*n* = 10), or more than three (*n* = 8) of the seven QUADAS-2 bias/applicability categories (see Supplementary Table 1).

Included studies (*n* = 37) reported on 1,599 unique patients [747 stroke; 13 intracranial hemorrhages; 26 other central causes (including vestibular migraine, multiple sclerosis, brainstem encephalitis); 743 acute unilateral vestibulopathy; 6 benign paroxysmal positional vertigo (BPPV) and 64 other], full study details are provided in Supplementary Table 2. Eleven studies reported on mixed populations with both peripheral and central causes, whereas the other 26 studies were focusing on either cAVS patients (*n* = 22) or pAVS patients (*n* = 4) only. The study design was cross-sectional in all but 3 studies and data collection was retrospective in the majority (*n* = 23 studies). Bedside testing was performed most frequently by expert neuro-otologists (17 out of 37 studies), but various other specialists were involved as well in other studies (see Table 1 for details). Information on whether SN was assessed with fixation preserved or suppressed (e.g., by use of Frenzel goggles) was provided in 13 manuscripts only, with fixation suppressed in two studies, fixation preserved in four studies, and examinations performed both with and without fixation in seven studies.

**Table 1:** Key epidemiologic aspects.

	**cAVS**	**pAVS**
**Gender**		
Women	286	373
Men	490	401
Not reported	10	39
Total	786	813
**Diagnoses**		
Ischemic stroke	747	NA
PICA territory	220	NA
AICA territory	67	NA
SCA territory	13	NA
Brainstem	209	NA
Not specified	238	NA
Hemorrhagic stroke	13	NA
Vestibular migraine	13	NA
Other^*^	13	NA
Acute vestibular neuropathy	NA	743
Menière's disease	NA	17
Labyrinthitis	NA	26
BPPV	NA	6
SSNHL with vestibulopathy	NA	7
Vestibular schwannoma	NA	3
Unclear	NA	11
Study design	N [Studies, subjects]	
Prospective cross-sectional	[14, 714]	
Retrospective cross-sectional	[20, 541]	
Retrospective longitudinal	[3, 344]	
**Study target group**		
cAVS population	[22, 409]	
pAVS population	[4, 353]	
Mixed cAVS and pAVS population	[11, 837]	
**Assessment of SN—setup**		
Fixation preserved	[4, 270]	
Fixation suppressed	[2, 319]	
Both with fixation preserved and fixation suppressed	[7, 208]	
NR	[24, 802]	
**Assessment of SN—examiner**		
ED physicians	[2, 48]	
ENT specialists	[7, 430]	
Neurologists	[8, 239]	
Experienced neuro-otologists	[17, 676]	
Neurology residents	[1, 114]	
Unclear	[2, 92]	
**Assessment of SN—vertical/torsional plane** ^†^		
Only horizontal SN assessed and reported	[8, 362]	
Both horizontal and vertical/torsional SN assessed and reported^‡^	[26, 1,188]	
Both horizontal and vertical/torsional SN assessed, patients with vertical/torsional SN excluded	[3, 49]	

AICA, anterior inferior cerebellar artery; AVS, acute vestibular syndrome; BPPV, benign paroxysmal positional vertigo; cAVS, central AVS; ENT, ear nose throat; pAVS, peripheral AVS; PICA, posterior inferior cerebellar artery; SCA, superior cerebellar artery; SN, spontaneous nystagmus; SSNHL, sensorineural hearing loss.

^*^Other central causes included multiple sclerosis (n = 7), brainstem encephalitis (n = 1), cerebellar metastases (n = 1), Wernicke encephalopathy (n = 1), paraneoplastic (n = 2), carbamazepine intoxication (n = 1).

^†^For studies that did not provide any information on the assessment of torsional and vertical SN, the corresponding author was contacted. Whereas, two corresponding authors provided additional information confirming incomplete or no assessment of vertical/torsional SN (24, 25), it remained unclear in four studies whether an assessment of vertical and torsional SN was performed (26–29). Those studies were excluded from the analysis of vertical/torsional SN patterns and frequency.

^‡^Note that two of these 26 studies reported on quantitative SN analysis only (30, 31), thus patients from Choi et al. (n =8) and from Nham et al. (n = 205) were excluded for the assessment of bedside SN characterization.

While in some studies results from both the initial bedside oculomotor examination and a subsequent dedicated quantitative oculomotor examination are reported, we focused on the findings from the clinical bedside oculomotor exam. In two studies initially included, no bedside testing could be identified, thus these studies were excluded from further analysis (30, 31). In another study included it remained unclear if reported qualitative spontaneous nystagmus patterns were based on bedside testing or on quantitative VOG recordings (32).

### 3.1. Distribution and diagnostic accuracy of spontaneous nystagmus patterns

Taking into account all 35 studies reporting on bedside SN characteristics in either plane, a SN was observed in almost all pAVS patients and in about half of all cAVS patients [684/701 (97.6%) vs. 293/672 (43.6%)]. Overall, 31 studies did not require presence of a horizontal SN as an inclusion criterion. One of those 31 studies was excluded as 3 out of 4 cells were empty (33). In the remaining 30 studies, absence of any horizontal SN was more frequently observed in cAVS patients than in pAVS patients (350/634 [55.2%] vs. 28/398 [7.0%], *p* < 0.001). Isolated torsional (12/555, 2.2%) or isolated vertical SN (27/555, 4.9%) was noted in very few participants only, but again was significantly more often found in cAVS patients than in pAVS patients (*p* < 0.001; see Table 2 for details).

**Table 2:** Nystagmus patterns in AVS patients with SN (including only those 24 studies that reported on SN in all planes)^*^.

**Beating direction of SN**	**cAVS (*n*, %)**	**pAVS (*n*, %)**	**Statistical analysis (Fisher's exact test)**	**Comments**
**Horizontal**				
Horizontal only	202, 36.4%	314, 74.8%	*p* < 0.001	
Horizontal-torsional	29, 5.2%	87, 20.4%	*p* < 0.001	
Horizontal-vertical	18, 3.2%	0, 0.0%	*p* < 0.001	
Horizontal-vertical-torsional	10, 1.8%	1, 0.2%	*p* = 0.029	
Any horizontal nystagmus	259, 46.7%	402, 95.7%	*p* < 0.001	
**No horizontal nystagmus**				
Torsional-vertical	17.3.1%	10, 2.4%	*p* = 0.56	All peripheral cases were patients with inferior vestibular neuritis
Torsional only	12, 2.2%	0, 0.0%	*p* < 0.001	
**Vertical only**				
DBN only	8, 1.4%	0, 0.0%	*P* = 0.012	
UBN only	18, 3.2%	0, 0.0%	*p* < 0.001	
Vertical only (dir ns)	1, 0.2%	0, 0.0%	*p* = 1.00	
Vertical only (all)	27, 4.9%	0, 0.0%	*p* < 0.001	
All patients (from 24 selected studies)	555, 100%	420, 100%		

^*^Data from 24 studies was included (8, 9, 32, 34–54).

Eleven studies reported on horizontal SN only, with vertical/torsional SN being either considered an exclusion criterion (33, 55, 56), focusing on the reporting of horizontal SN (24, 25, 57, 58), or not providing any information on whether patients were assessed for the presence/absence of vertical and torsional SN (27–29, 37). These manuscripts were excluded from all analyses concerning the prevalence and distribution of vertical/torsional SN in AVS patients. The remaining 26 studies reported on SN in all planes (horizontal, vertical, and torsional). From those 26 studies, two studies only provided quantitative SN data (30, 31), leaving 24 studies and 975 patients (pAVS = 420; cAVS = 555) for the analysis of bedside SN in all three planes. Focusing on those 24 studies that reported on SN patterns in all planes, a horizontal or horizontal-torsional SN was significantly more often found in pAVS than in cAVS patients (401/420 [95.5%] vs. 231/555 [41.6%], *p* < 0.001), isolated torsional, isolated torsional-vertical and/or vertical SN patterns were more prevalent in cAVS than in pAVS (84/555 [15.1%] vs. 11/420 [2.6%], *p* < 0.001; see Table 2 for details).

**Figure 2:**
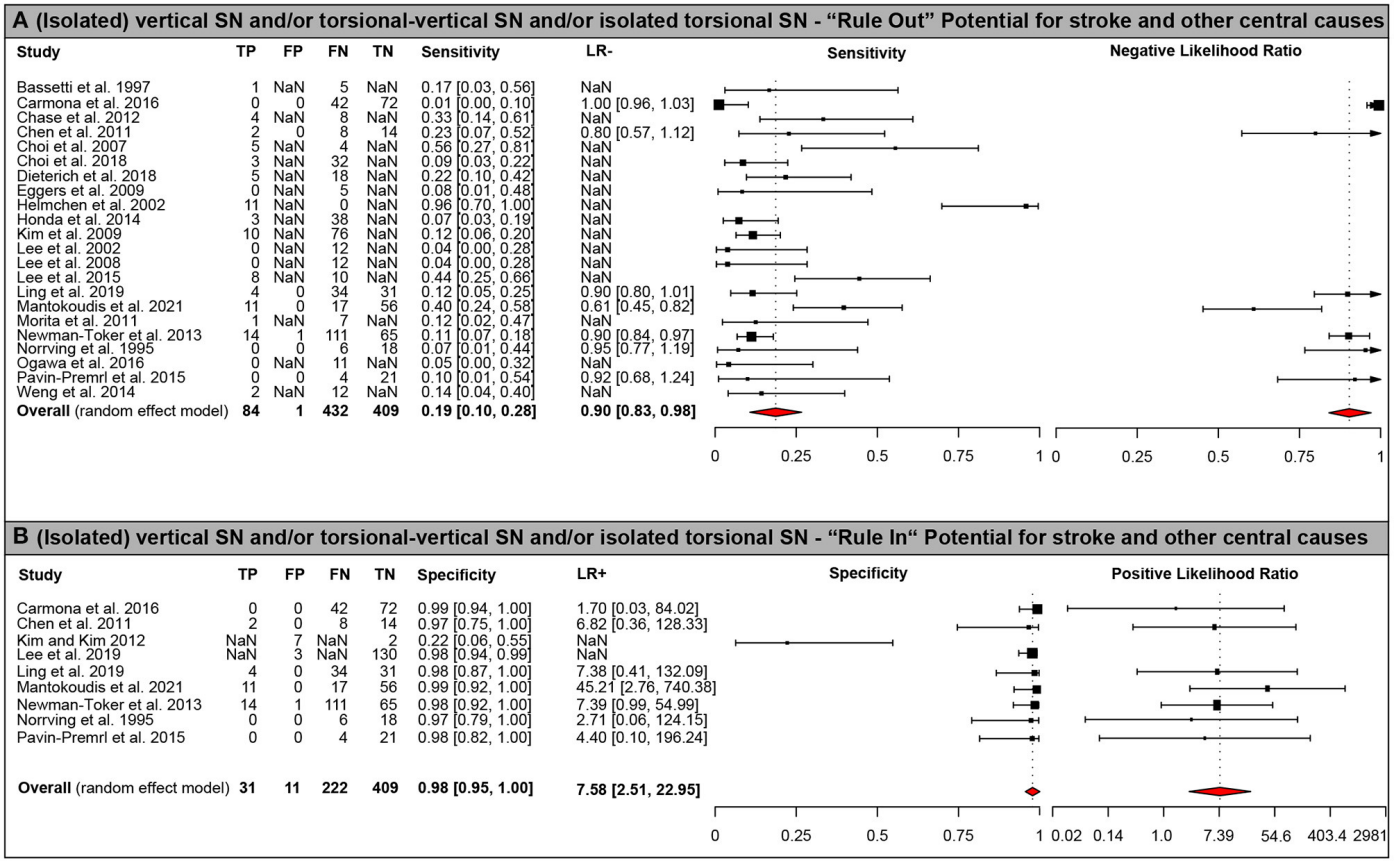
Forest plots of diagnostic test properties. Forest plots of diagnostic test properties for an (isolated) vertical/vertical-torsional or torsional SN. **(A)** sensitivity and negative likelihood ratio (LR-) including 95% confidence intervals (CI); **(B)** specificity and positive likelihood ratio (LR+) including 95% CI. Single study results and aggregated values for all test properties (including 95% CI) are provided for all studies included (*n* = 25). Summary measures were calculated using a random effects model using the DerSimonian-Laird estimator (20, 21). Heterogeneity amongst all studies was significant both for ruling out (i.e., sensitivity, *p* < 0.001) and for ruling in (i.e., specificity, *p* < 0.001) central (mostly vertebrobasilar ischemic stroke) causes using Cochran's Q. Note that the axis for positive likelihood ratio uses an exponential scale.

Diagnostic accuracy of SN patterns for predicting a central origin varied significantly with the SN beating pattern used. For an (isolated) vertical/vertical-torsional SN or an isolated torsional SN, specificity (97.7% [95% CI = 95.1–100.0%]) for a central origin etiology was high, whereas sensitivity (19.1% [10.5–27.7%]) was low (see Table 3 and Figure 2 [forest plots]). When being slightly more inclusive, i.e., using a SN beating pattern of vertical, torsional, vertical-torsional SN with or without horizontal SN, sensitivity was somewhat higher (19.8% [10.7–28.9%]) and specificity was identical (97.7% [95.1–100.0%]). When using more restrictive SN beating patterns, sensitivity further decreased, whereas specificity remained very high. This was true both for selecting a vertical SN pattern (with or without an accompanying horizontal SN; sensitivity = 12.9% [7.6–18.3%]; specificity = 98.2 [96.2–100.0%]) and an isolated torsional SN pattern (sensitivity = 1.0% [0.0–1.8%]; specificity = 99.4 [98.6–100.0%]). Absence of a horizontal SN was highly predictive for a central cause (specificity = 89.8% [84.3–95.4%]), whereas sensitivity was moderate only (sensitivity = 51.6% [3,920–64.1%]; see Table 3 for details).

**Table 3:** Diagnostic accuracy of different SN patterns in AVS.

**SN pattern**	***N* (studies)**	***N*** **(subjects)**		**Sensitivity % (95% CI)**	**LR- (95% CI)**	**Specificity % (95% CI)**	**LR+ (95% CI)**
	**Total, pAVS, cAVS**	**pAVS**	**cAVS**				
(Isolated) vertical SN and/or torsional-vertical SN and/or isolated torsional SN^*^	24, 9, 22	420	555	19.1 (10.5–27.7)	0.90 (0.83–0.98)	97.7 (95.1–100.0)	7.59 (2.51–22.95)
Spontaneous vertical, torsional or vertical-torsional *N* (w/wo hor SN)^*^	24, 9, 22	420	555	19.8 (10.7–28.9)	0.89 (0.83–0.96)	97.7 (95.1–100.0)	8.96 (3.05–26.31)
Spontaneous vertical *N* (w/wo hor SN)^*^	24, 9, 22	420	555	12.9 (7.6–18.3)	0.92 (0.86–1.00)	98.2 (96.2–100.0)	6.55 (2.13–20.11)
Spontaneous torsional *N* only^*^	24, 9, 22	420	555	1.0 (0.0–1.8)	1.00 (0.98–1.00)	99.4 (98.6–100.0)	2.54 (0.66–9.78)
NO spontaneous horizontal *N*^§^	30, 11, 26	396	634	51.6 (39.2–64.1)	0.55 (0.39–0.77)	89.8 (84.3–95.4)	6.33 (2.52–15.90)

^*^Data from 24 studies were included (8, 9, 32, 34–54).

^§^Data from 30 studies were included (8, 9, 24–27, 29, 32, 34–53, 55, 57), whereas one additional study was excluded as three cells were zero (33).

CI, confidence interval; hor, horizontal; LR, likelihood ratio; N, nystagmus; SN, spontaneous nystagmus.

### 3.2. Distribution of spontaneous nystagmus patterns and relation to lesion location

Amongst all patients with SN, the beating direction strongly depended on the underlying cause (peripheral vs. central) and the lesion location for central causes. For central lesions, the distribution of SN patterns varied amongst different affected vascular territories and anatomical lesion locations as shown in Supplementary Table 3. Specifically, the vast majority of PICA strokes (77/89) presented with horizontal only SN, whereas for AICA strokes a horizontal-torsional SN was found at the frequency than a horizontal only SN (20 cases each). Looking at different brainstem strokes, mesencephalic lesions and pontine lesions resulted in various SN patterns. In contrast, the SN pattern critically depended on the type of medullary lesion. Whereas, medial medullary lesions demonstrated mostly with horizontal SN only or UBN only patterns (38/43 patients), lateral medullary strokes presented with a combined horizontal-torsional-vertical SN or a purely horizontal SN at similar rates (36 vs. 40%). Compared to AICA strokes, fractions of patients with any SN were significantly smaller in PICA strokes (65.0 vs. 82.0%, *p* = 0.031), but not in SCA strokes (62.5 vs. 82.0%, *p* = 0.34). At the level of the medulla oblongata, medial medullary strokes presented with any SN less often than (dorso-)lateral medullary strokes (44.8 vs. 80.6%, *p* < 0.001).

### 3.3. Spontaneous nystagmus beating direction in relation to lesion location

SN beating direction in relation to the type and location of the underlying disease could be retrieved in a total of 1,062 patients originating from 35 studies (cAVS = 378, pAVS = 684). Ipsilesional and contralesional beating directions of horizontal (or torsional) SN in cAVS were found at similar frequency (106/378 [28.0%] vs. 82/378 [21.7%], *p* = 0.052), whereas for pAVS a contralesional SN was significantly more frequent (651/684 [95.2%] vs. 17/684 [2.5%], *p* < 0.001). Noteworthy, beating direction in relation to the lesion was not reported in 41.5% of all cAVS patients, whereas this fraction was much smaller in pAVS patients (2.3%). Furthermore, no assessment of SN beating direction in relation to the lesion location was possible in those cAVS cases with bilateral lesions (*n* = 10), non-focal lesions (*n* = 8), or purely vertical SN patterns (*n* = 15), as shown in Table 4.

**Table 4:** Distribution of SN beating direction (*n* = 1,062) in pAVS and cAVS patients.

**Lesion type and beating direction**	**cAVS (*n*, %)**	**pAVS (*n*, %)**
**Lateralized focal lesions**		
Ipsilesional SN beating direction	106, 28.0%	17, 2.5%
Contralesional SN beating direction	82, 21.7%	651, 95.2%
Bilateral focal lesions	10, 2.6%	0, 0.0%
Non-focal lesions^*^	8, 2.1%	0, 0.0%
Purely vertical SN^†^	15, 4.0%	0, 0.0%
Beating direction not reported	157, 41.5%	16, 2.3%
Total	378, 100%	684, 100%

^*^No focal lesions could be identified in 8 patients with vestibular migraine (56).

^†^One patient with a right-sided dorsolateral medullary stroke (50) presented with a torsional and downbeating spontaneous nystagmus with opposite torsional directions in both eyes (CW for left eye, CCW for right eye), thus beating direction of SN could not be determined.

When focusing on cAVS patients with SN and lateralized and focal infratentorial (i.e., cerebellar or brainstem) lesions, the lesion location had a significant impact on the SN beating direction patterns observed. Specifically, for PICA strokes presenting with horizontal (or torsional) SN, beating direction was ipsilesional more often than contralesional (26/109 [23.9%] vs. 7/109 6.4%], *p* = 0.006), while the opposite was observed for AICA strokes (1/46 [2.2%] vs. 29/46 [63.0%], *p* < 0.001) as shown in Table 5. Numbers for SCA lesions or combined PICA-AICA and PICA-SCA lesions were too small to reveal statistical significances. For lesions restricted to the medulla oblongata, the lesion location had a critical impact on the SN beating direction. Whereas, in those patients with lesions of the medial parts of the medulla an ipsilesional beating direction of SN was significantly more often observed than a contralesional beating direction (29/43 [67.4%] vs. 2/43 [4.7%], *p* < 0.001), the opposite was noted in patients with lesions of the (dorso-)lateral medulla oblongata (7/26 [26.9%] vs. 15/26 [57.7%], *p* = 0.048). For pontine or mesencephalic lesions, ipsilesional and contralesional SN beating directions were observed at similar frequencies.

**Table 5:** Distribution of SN beating direction in cAVS patients with focal lateralized brainstem and/or cerebellar lesions.

	**Ipsilesional (*n*, %)**	**Contralesional (n, %)**	**Comparison of relative frequency of occurrence (Fisher's exact test)**	**Purely vertical SN patterns (*n*, %)**	**Beating direction/lesion location NR (*n*, %)**	**Total**
**Specific vascular territories**						
PICA	26, 23.9%	7, 6.4%	***p*** **=** **0.006**	0, 0.0%	76, 58.5%	130
AICA	1, 2.2%	29, 63.0%	***p*** ** < 0.001**	0, 0.0%	16, 30.8%	52
SCA	1, 25.0%	0, 0.0%	*p* = 1.00	0, 0.0%	3, 60.0%	5
PICA-AICA	1, 25.0%	3, 75.0%	*p* = 0.32	0, 0.0%	0	5
PICA-SCA	2, 100.0%	0, 0.0%	*p* = 1.00	0, 0.0%	0	2
**Anatomical description only**						
Mesencephalon	5, 31.3%	9, 56.3%	*p* = 0.13	2, 12.5%	0, 0.0%	16
Pons	4, 44.4%	2, 22.2%	*p* = 1.00	0, 0.0%	3, 33.3%	9
**Medulla oblongata**						
Medial medulla	29, 67.4%	2, 4.7%	***p*** **<** **0.001**	7, 16.3%	5, 11.6%	43
(dorso-)lateral medulla	7, 26.9%	15, 57.7%	***p*** **=** **0.048**	1, 3.8%	3, 11.5%	26
Not further specified	5, 71.4%	1, 14.3%	*p* = 0.24	0, 0.0%	1, 14.3%	7
Brainstem and/or cerebellum	23, 30.3%	10, 13.2%	*p* = 0.12	11, 11.8%	40, 43.0%	93
Total	104, 30.4%	78, 22.8%		13, 3.8%	147, 43.0%	342

Bold values refer to statistically significant differences (Fisher's exact test).

### 3.4. The effect of fixation suppression on spontaneous nystagmus of peripheral and central origin

Only eight out of 35 studies reported on SN patterns both with fixation and without fixation at the bedside. Whereas, in six studies all patients were evaluation under both conditions (32, 36, 38, 49, 50, 54), in the remaining two studies only in a subset of patients SN was assessed both with fixation preserved and removed (8, 51). The effect of fixation on SN was reported in five studies only (36, 38, 50, 51, 54). Another study reported on SN fixation suppression only after caloric irrigation (32) and additional two studies did not provide any information on the effect on fixation suppression in those patients assessed in both conditions (8, 49).

In four studies, the effect of fixation suppression was reported qualitatively only. In a case series of 18 patients with dorso-lateral medullary stroke, 13 out of 18 patients showed an increase in horizontal–torsional SN with removal of visual fixation (qualitatively, at the bedside) (50). In another case series reporting on patients with (dorso-)lateral medullary infarction, the horizontal and vertical components of the SN were found to be markedly suppressed by visual fixation, whereas the authors noted that the torsional component was less effectively suppressed (38). In patients with mesencephalic stroke and predominantly torsional SN, the authors noted no increase of SN in darkness or with Frenzel goggles (36). In another case series with both pAVS and cAVS patients, fixation suppression of SN was found in eight of the 11 vestibular neuritis patients (73%) where fixation-suppression was tested. In the single stroke patient (PICA stroke) assessed, no fixation suppression of SN was found (51).

In two studies the impact of visual fixation removal was assessed quantitatively. Mantokoudis and co-workers reported a complete nystagmus fixation suppression in 49.5% of patients with AVS, with fractions in patients with vestibular neuritis being lower than in patients with vestibular strokes (40 vs. 62.5%). Whereas, the ocular fixation index (OFI) score calculated had no predictive value for detecting strokes, a nystagmus reduction of < 2°/s was linked to a central cause of AVS, with a sensitivity of 62.2% and a specificity of 84.8% in detecting a stroke (resulting in an overall diagnostic accuracy of 76.9% (95% confidence interval = 59–89%) (54). In another study mean horizontal nystagmus slow-phase velocity (SPV) with fixation in posterior circulation stroke patients (1.6 ± 2.5°/s, range 0–9.6°/s) was significantly slower than in acute vestibular neuritis patients (6.4 ± 5.2°/s, range 0–28.3°/s; *p* < 0.001) (31). In this study also a greater increase in nystagmus SPV with visual fixation suppression in acute vestibular neuritis compared to posterior circulation stroke was reported, with the mean absolute nystagmus SPV difference between no visual fixation and fixation conditions being significantly higher in acute vestibular neuritis patients compared to posterior circulation stroke patients (5.9 ± 4.7°/s vs. 0.7 ± 2.3°/s, *p* < 0.001).

### 3.5. The time course of spontaneous nystagmus recovery in peripheral and central AVS patients

Few studies provided a longitudinal assessment of SN characteristics in AVS patients. In one study the authors reported a significant difference in the duration of SN in acute vestibular neuritis patients depending on the extent of canal paresis in caloric irrigation. Specifically, a significantly faster recovery from SN was noted in patients with < 25% canal paresis compared to those patients with more profound canal paresis (i.e., >25%; SN duration: 3.0 ± 1.7 days vs. 4.2 ± 1.9 days, *p* < 0.001) (28). In another study, time to SN remission was significantly (*p* < 0.05) shorter in patients with isolated involvement of the inferior branch of the vestibular nerve (inferior vestibular neuritis; SN remission time = 10.3 ± 5.9 days) compared to patients with involvement of either the superior branch of the vestibular nerve (superior vestibular neuritis, 21.5 ± 6.9 days) or both the inferior and the superior branch (total vestibular neuritis, 21.0 ± 13.4 days) (53). In a study reporting on 10 patients with cAVS (and SN in 4/10) initially misdiagnosed as being of peripheral origin, SN duration was reported in three out of four patients only. Specifically, SN disappeared after 1 or 2 days in two patients, whereas it was found to be persistent in another patient (52).

## 4. Discussion

In this systematic review and meta-analysis, we addressed the diagnostic accuracy of spontaneous nystagmus (SN) patterns in patients with acute vestibular syndrome (AVS) of either peripheral (pAVS) or central (cAVS) origin. A SN was observed in almost all pAVS patients and in about half of all cAVS patients (97.6 vs. 43.6%). In those AVS patients demonstrating a SN (with fixation preserved or removed), the SN pattern varied depending on the underlying cause. While a horizontal or horizontal-torsional SN was significantly more often found in pAVS than in cAVS patients (94.8 vs. 43.4%, *p* < 0.001), isolated torsional, isolated torsional-vertical and/or vertical SN patterns were more prevalent in cAVS than in pAVS (15.1 vs. 2.6%, *p* < 0.001). When present, it was highly predictive for a central cause (specificity = 97.7% [95% CI = 95.1–100.0%]). The SN beating direction was contralesional in the vast majority of pAVS cases (95.2%), whereas in cAVS cases with focal, lateralized lesions fractions of patients with ipsilesional and contralesional beating direction were similar (28.0 vs. 21.7%, *p* = 0.052). Thus, in cAVS patients the SN beating direction itself does not allow a prediction on the lesion side. However, for given lesion locations including the medulla oblongata and certain vascular patterns (PICA strokes, AICA strokes), a predominance of either ipsilesional or contralesional horizontal/torsional SN was noted.

### 4.1. The diagnostic accuracy of spontaneous nystagmus patterns and its distribution in relation to the lesion location

In those 24 studies (reporting on a total of 975 patients) providing information on SN beating direction in any plane (horizontal, vertical and torsional), a vertical and/or torsional SN (with or without an accompanying horizontal SN component) was found in a minority (20.4%) of cAVS patients only. When excluding those patients with a horizontal SN component in addition to the presence of a torsional or torsional-vertical nystagmus (i.e., focusing on isolated torsional, isolated torsional-vertical and vertical SN patterns), such a pattern was found in 15.1% of all cAVS patients. While its sensitivity (for ruling out stroke) was low (19.1% [10.5–27.7%]), its specificity (i.e., for ruling in stroke) was very high (97.7% [95.1–100.0%]). Noteworthy, most of the few false-positive cases were linked to inferior vestibular neuritis (IVN), resulting in a combined torsional-downbeating (contralesional) SN pattern, as reported by two studies in our systematic review (49, 53). In IVN, vestibular deficits may be accompanied by ipsilesional, new-onset hearing loss as well. Furthermore, in these patients the horizontal head-impulse test may be normal, resulting in a “central” HINTS pattern (7) and even when adding a fourth sign to the HINTS [new-onset unilateral hearing loss, HINTS “plus” (8)], the pattern may be interpreted as being of central origin. For such cases, obtaining a quantitative video-head-impulse test for all six canals is crucial (23, 59), as it allows canal-specific testing of the vestibulo-ocular reflex (60) and thus should demonstrate isolated impairment of the posterior semicircular canal in case of IVN (49).

In the clinical assessment and in combination with other subtle oculomotor findings, the presence of a horizontal SN indeed may provide valuable. For example, a horizontal/horizontal-torsional SN beating toward the side demonstrating an abnormal head-impulse test points to a central cause (e.g., due to an AICA stroke) rather than to an acute peripheral vestibulopathy (expecting the SN to beat away from the side with the deficient head-impulse test).

Importantly, even in those studies that reported on SN with fixation removed, there was a significant fraction of cAVS patients that did not demonstrate any SN, with fractions in the range of 33–63% (9, 32, 38). Thus, the absence of any SN in a patient with prolonged acute vertigo or dizziness meeting diagnostic criteria for AVS is highly predictive of a central cause with a specificity of 97.9% (95% CI = 96.9–98.9%) and should be considered a central sign in this clinical situation as emphasized by (31).

In patients with underlying central causes of AVS, the SN pattern varied depending on the lesion location, emphasizing distinct contributions to ocular stability by different brainstem and cerebellar structures. Specifically, a torsional component of the SN was observed more frequently in AICA strokes than in PICA strokes and in lateral medullary lesions than in medial medullary lesions. For a detailed discussion of the anatomical-nystagmus correlation with regards to the vascular territory and the lesion sites as observed in the literature, we would like to refer to a recent publication by Nham and colleagues (31).

### 4.2. The impact of focal lateralized lesions on SN beating patterns in cAVS

For peripheral-vestibular lesions, by far most often an acute vestibular neuropathy (vestibular neuritis) was diagnosed (743/813, 91.4%). In these cases, a contralesional nystagmus, i.e., with the fast-phase of the SN beating away from the affected ear, is observed (13). This was reflected in the distribution of pAVS cases, with 95% demonstrating a contralesional SN pattern. In contrast, the lateralizing value of a horizontal/horizontal-torsional SN in central lesions is limited. Overall, patients with focal, lateralized lesions demonstrated ipsilesional and contralesional horizontal/torsional SN beating directions in similar fractions (28.0 vs. 21.7%, *p* = 0.052). Thus, in cAVS patients the SN beating direction itself does not allow a prediction on the lesion side. However, for given lesion locations such as medial or (dorso-)lateral medullary strokes and certain vascular patterns (PICA strokes, AICA strokes), a predominance of either ipsilesional or contralesional horizontal/torsional SN was noted in our systematic review. In combination with other focal neurologic findings (e.g., pointing to a dorsolateral medullary stroke), the SN beating direction may be valuable in confirming the side of the lesion in these patients. A further differentiation of the beating direction in PICA strokes has been discussed by Nham and colleagues (31) using quantitative SN measurements, with an ipsilesional horizontal SN in those PICA strokes affecting the cerebellum only and a contralesional beating direction in those PICA strokes with brainstem involvement only. Noteworthy, for our meta-analysis we could not retrieve more detailed information on the lesion pattern (brainstem vs. cerebellum) in PICA strokes. With an overall preferentially ipsilesional SN pattern in PICA strokes in our dataset, this would suggest that the majority of PICA stroke cases included here were restricted to the cerebellum.

### 4.3. Fixation suppression of spontaneous nystagmus is more pronounced in pAVS than in cAVS cases

A decrease (or even cessation) of SN by allowing the acutely dizzy patient to fixate and an enhanced SN by removal of visual fixation is considered an important finding in peripheral-vestibular causes such as acute VN (13). In contrast, a lacking impact of fixation on SN intensity has been associated with central causes in neurology textbooks (61), whereas a systematic review of the literature has emphasized lacking evidence on the accuracy of such a clinical suppression sign (15). More recent publications identified in this systematic review now provide a more detailed picture. Quantitative data on the impact of visual fixation removal was available from two studies, demonstrating that a complete nystagmus fixation suppression may be observed in both patients with acute peripheral vestibulopathy and with vestibular strokes at similar frequency (40 vs. 62.5%) (54). Importantly, the magnitude of nystagmus reduction was helpful in distinguishing peripheral from central causes. Specifically, a nystagmus reduction of < 2°/s was linked to a central cause of AVS (sensitivity = 62.2%, specificity = 84.8%) in one study (54). In another study, a greater increase in nystagmus slow-phase velocity (SPV) with visual fixation suppression in acute vestibular neuritis compared to posterior circulation stroke was reported (5.9 ± 4.7°/s vs. 0.7 ± 2.3°/s, *p* < 0.001) (31). Noteworthy, in this study the mean horizontal nystagmus SPV with fixation was significantly slower in posterior circulation stroke patients than in acute vestibular neuritis patients (1.6 ± 2.5°/s vs. 6.4 ± 5.2°/s, *p* < 0.001) (31).

At the bedside, upon removal of visual fixation an increase in horizontal-torsional SN intensity was noted in stroke patients with (dorso-)lateral medullary lesions (50). Noteworthy, the effect of visual fixation seems to depend on the SN plane, as observed in another study with dorso-lateral medullary stroke patients (38). In this study, the horizontal and vertical components of the SN were found to be markedly suppressed by visual fixation, whereas the authors noted that the torsional component was less effectively suppressed (38). A minor impact of visual fixation on torsional SN was also reported in patients with mesencephalic stroke and predominantly torsional SN; specifically the authors noted no increase of SN in darkness or with Frenzel goggles (36).

In summary, the value of SN fixation suppression for distinguishing peripheral from central AVS patients is limited and usually requires a quantitative (VOG-based) assessment of the SN-pattern (54). At the bedside, a qualitative assessment may be not sensitive enough to depict differences in magnitude of response to fixation and fixation removal. When examining the impact of visual fixation removal in this setting, however, clinicians should focus on changes in horizontal and/or vertical SN intensity. Importantly, the presence of fixation suppression does not rule out a central cause of AVS as emphasized by Mantokoudis and co-workers (54).

### 4.4. The time course of SN in AVS patients

In this systematic review few included studies reported on serial SN assessments, providing information on the time course of SN. Two of those three studies that did so focused on vestibular neuritis patients (49, 53), whereas in only very few cAVS patients from a single study serial SN assessments were obtained (52). Thus, it remains unclear to which extent delayed presentation to the ED impacts the diagnostic accuracy of SN pattern assessment.

### 4.5. Limitations

This study has several limitations that need to be further discussed. First, only a minority of studies reported on relatively unselected acutely dizzy patient populations, whereas a majority of studies was at increased risk for a selection bias by including subpopulations such as medial medullary strokes or mesencephalic strokes only, potentially overestimating the presence of certain SN patterns. Over 90% of all peripheral-vestibular cases included were diagnosed with an acute vestibular neuritis, whereas other diagnoses such as Menière's disease which may present with distinct SN patterns (including an irritative ipsilesional SN) were scarce. Not all studies provided specific numbers on delay from symptom onset to clinical examination of spontaneous nystagmus at the bedside and a subset of patients may have received delayed (i.e., after more than 72 h) examination only. While we excluded single patients that received oculomotor bedside testing delayed, this information was not available in all studies. When aggregated numbers were provided, we required that at least 75% of patients received SN evaluation within 72 h after symptom onset. Furthermore, we assumed that patients with obvious stroke symptoms such as hemiparesis or hearing loss generally present to the ED within 24 h after symptom onset and will receive initial clinical examination within hours after presentation to the ED. This pragmatic approach reflects real-life conditions. Note that by potentially including a subset of patients with subacute presentation, spontaneous nystagmus may have disappeared already, thus will result in an underestimation of sensitivity and specificity of SN.

Second, the clinical background of the assessing physicians varied significantly, ranging from subspecialist neuro-otologists to general neurologists or ENT-specialists and emergency physicians. Thus, the level of granularity in describing SN patterns and the amount of expertise likely varied from study to study and physicians not appropriately trained in characterizing a SN may have missed more subtle SN patterns (such as e.g., isolated torsional SN). Third, we did not analyze nystagmus at different gaze positions, which may provide useful also in distinguishing peripheral from central type nystagmus (e.g., by assessing whether the nystagmus follows Alexander's law or not or whether the nystagmus is encoded in a head-fixed or an eye-fixed reference frame). Fourth, the description of lesion locations in relation to the vascular territory affected and/or the definitions of anatomical regions of the brain involved varied amongst studies. This led to more clustered subgroups with both lesion locations based on the vascular supply and the anatomical lesion site, potentially overshading more general patterns, e.g., with regards to the beating direction of SN. Fifth, data on the time course of SN recovery were scarce, thus no conclusions on the time course of SN especially in cAVS patients could be drawn. Sixth, there is a variety in the equipment used for bedside qualitative nystagmus assessments (e.g., Frenzel glasses or videofrenzels) with differences in the sensitivity of detecting nystagmus. For example, the sensitivity in detecting nystagmus with Frenzel glasses is lower than with video frenzels (62).

## 5. Conclusions

Unlike other subtle oculomotor bedside findings (such as testing for gaze-evoked nystagmus, vertical divergence, the integrity of the vestibulo-ocular reflex and new-onset unilateral hearing loss), which allow a differentiation between a peripheral and a central cause with high diagnostic accuracy [HINTS+-sensitivity = 99.2% [96.1–100.0%], specificity = 97.0% [90.4–99.5%] (8)], the interpretation of the SN pattern observed in acutely dizzy patients is less straight-forward. Whereas, in those patients that present with an (isolated) vertical and/or torsional SN, a central cause is highly likely (Specificity = 97.7% [95.1–100.0%]) and absence of a horizontal SN (even with fixation removed) strongly suggests a central origin of the patient's AVS (Specificity = 89.8% [84.3–95.4%]), the presence of a horizontal or horizontal-torsional SN does not allow a distinction between a peripheral or a central cause. Furthermore, such an (isolated) vertical and/or torsional SN pattern is found in a minority (15.1%) of central AVS patients only, as reflected by a low sensitivity (19.1% [10.5–27.7%]) and a combined torsional-downbeating SN pattern may be observed in peripheral AVS also in cases with isolated lesions of the inferior branch of the vestibular nerve. Furthermore, the lateralizing value of a horizontal and/or torsional SN in suspected focal lesions is limited at the bedside. While certain lesion locations (anatomical or vascular territory) are associated more often with an ipsilesional or contralesional SN, this cannot be generalized. Additionally, presence of visual fixation suppression does not rule out a central cause of AVS.

In conclusion, a detailed characterization and documentation of the SN pattern observed initially is essential. With a majority of such patients presenting to the emergency department, dedicated training of frontline providers including ED-physicians and neurology/ENT residents in describing and interpreting SN patterns is of great importance.

## Data availability statement

The original contributions presented in the study are included in the article/Supplementary material, further inquiries can be directed to the corresponding author.

## Author contributions

MW coded abstract and full-text studies, helped in the analysis, and the interpretation of the data. ZW conducted all analysis and oversaw the interpretation. CM and GM helped abstracting the data and analyzing the results. SC helped develop study protocols, helped abstracting the data, and analyzing the results. AT performed or directly oversaw all aspects of study from conception through completion (principal investigator), designed and conducted the literature search strategy, coded abstract and full-text studies, led analysis, and interpretation of data. All authors critically reviewed and edited the manuscript, seen, and approved the final version.
