# Supplementary material for: Systematic review and meta-analysis of the diagnostic accuracy of spontaneous nystagmus patterns in acute vestibular syndrome
Source: Front Neurol. 2023 Jun 16;14:1208902. doi: 10.3389/fneur.2023.1208902 (PMC10312004; doi:10.3389/fneur.2023.1208902)
Supplement: Supplementary file 1 [file Data_Sheet_1.docx]

# **Appendices 1-3**

# **Appendix 1- electronic search strategy, coding-scheme for the systematic review and data analysis**

**The search strategy was designed by a neurologist with relevant domain expertise in neuro-otology (AAT). We searched MEDLINE and Embase for English-language articles, using the following strategies with the following components: (1) vertigo/dizziness, (2) diagnostic accuracy of spontaneous nystagmus as assessed at the bedside or by quantitative eye-movement recordings, and (3) acute vestibular syndrome (ischemic stroke, acute peripheral vestibulopathy). We also performed a manual search of reference lists from eligible articles and contacted corresponding authors where necessary. We did not seek to identify research abstracts from meeting proceedings or unpublished studies.**

AGGREGATED/COMPOSITE VERSION (September 1st, 2022) [PubMed ~3668 abstracts; EMBASE 3228 abstracts]

((dizz*[tiab] OR vertigo[tiab] OR vestibular[tiab]) AND (prodrom*[tiab] OR diagnos*[tiab] OR manifestation*[tiab] OR clinical feature*[tiab] OR symptom*[tiab] OR “physical examination”[mh] OR “medical history taking”[mh] OR physical exam*[tiab] OR professional competence[mh] OR “sensitivity and specificity”[tiab] OR “sensitivity and specificity”[mh] OR “reproducibility of results”[mh] OR “observer variation”[mh] OR “diagnostic tests, routine”[mh] OR “decision support techniques”[mh] OR “bayes theorem”[mh] OR dizziness/physiopathology[mh] OR vertigo/physiopathology[mh] OR dizziness/diagnosis[mh] OR vertigo/diagnosis[mh]) AND (acute peripheral vestibulopathy[tiab] OR labyrin*[tiab] OR vestibular neuritis[tiab] OR vestibular neuronitis[tiab] OR vestibular syndrome[tiab] OR cerebrovascular[tiab] OR stroke*[tiab] OR cerebellar[tiab] OR hemorrhag*[tiab] OR haemorrhag*[tiab] OR vertebrobasilar insufficiency[tiab] OR TIA[tiab] OR transient ischemic attack[tiab]) NOT (animals[mh] NOT humans[mh]) AND eng[la] AND 1980:2022[dp] NOT review[pt])

DISAGGREGATED VERSION [to demonstrate overall structure] (September 1st, 2022)

((dizz*[tiab] OR vertigo[tiab] OR vestibular[tiab])

AND

(prodrom*[tiab] OR diagnos*[tiab] OR manifestation*[tiab] OR clinical feature*[tiab] OR symptom*[tiab] OR “physical examination”[mh] OR “medical history taking”[mh] OR physical exam*[tiab] OR professional competence[mh] OR “sensitivity and specificity”[tiab] OR “sensitivity and specificity”[mh] OR “reproducibility of results”[mh] OR “observer variation”[mh] OR “diagnostic tests, routine”[mh] OR “decision support techniques”[mh] OR “bayes theorem”[mh] OR dizziness/physiopathology[mh] OR vertigo/physiopathology[mh] OR dizziness/diagnosis[mh] OR vertigo/diagnosis[mh])

AND

(acute peripheral vestibulopathy[tiab] OR labyrin*[tiab] OR vestibular neuritis[tiab] OR vestibular neuronitis[tiab] OR vestibular syndrome[tiab] OR cerebrovascular[tiab] OR stroke*[tiab] OR cerebellar[tiab] OR hemorrhag*[tiab] OR haemorrhag*[tiab] OR vertebrobasilar insufficiency[tiab] OR TIA[tiab] OR transient ischemic attack[tiab])

NOT (animals[mh] NOT humans[mh])

AND eng[la]

AND 1980:2022[dp]

NOT review[pt])

**For Embase:**

(dizz*:ab,ti OR vertigo:ab,ti OR vestibular:ab,ti) AND (prodrom*:ab,ti OR diagnos*:ab,ti OR manifestation*:ab,ti OR ‘clinical features’:ab,ti  OR ‘clinical feature’:ab,ti OR symptom*:ab,ti OR ‘physical examination’/de OR ‘anamnesis’/de OR ‘physical exam’:ab,ti OR 'professional competence'/de OR ‘sensitivity and specificity’:ab,ti OR **'**sensitivity and specificity**'**/de OR 'reproducibility'/de OR  'observer variation'/de OR  'diagnostic test'/de OR  'decision support system'/de OR 'Bayes theorem'/de) AND  (‘acute peripheral vestibulopathy’:ab,ti OR labyrin*:ab,ti OR ‘vestibular neuritis’:ab,ti OR ‘vestibular neuronitis’:ab,ti OR ‘vestibular syndrome’:ab,ti OR cerebrovascular:ab,ti OR stroke*:ab,ti OR cerebellar:ab,ti OR hemorrhag*:ab,ti OR haemorrhag*:ab,ti OR ‘vertebrobasilar insufficiency’:ab,ti OR tia:ab,ti OR ‘transient ischemic attack’:ab,ti OR ‘transient ischaemic attack’:ab,ti) AND [1-1-1980]/sd AND [humans]/lim AND [English]/lim AND ([article]/lim OR [article in press]/lim)

## **Inclusion and exclusion rules for abstracts & full-text manuscripts**

**All gathered literature was subject to title/abstract screening by two independent reviewers (AAT/MW). Abstract review coding rules are provided below. Full-text screening was applied to all citations considered eligible or possibly eligible by at least one reviewer. Two independent reviewers (AAT/MW) determined whether full-text manuscripts are eligible and, if not, provided a reason for exclusion (see full-text review coding rules below). Differences were resolved by discussion and consensus. MW and AAT completed a hand search of the reference lists of selected articles for additional citations. For citations identified by hand search, the full process was repeated iteratively until no additional manuscripts were found for inclusion. Inter-rater agreement on full-text inclusion was calculated using Cohen’s kappa.(Cohen, 1960)**

## **Abstract review coding rules**

1) Coding status options are “Yes”, “No”, “Maybe”. We will review full text of “Yes” and “Maybe”.

2) Err on the side of “Maybe” if there is doubt about a “No”; this is more conservative.

3) If there is only a title, exclude it only if you feel confident; otherwise code it as “Maybe”.

4) Each "No" should be coded with a reason for exclusion.

5) Reasons for exclusion are listed below 0-7. Go through them in order from 0 to 7 for each abstract, coding the first reason for exclusion only, not multiple reasons for exclusion. Only code "0" for “not English” if you are sure it is “not English”.

6) Two independent raters (AAT/MW) will code reason for exclusion, but we will *not* mandate agreement on exclusion reason at the abstract level.

7) Occasionally an abstract seems inappropriate for another reason. In such cases, code as “other”. There should be few abstracts coded as “other.”

## **Abstract reasons for exclusion**

| 0 | not English | manuscript is not in English |
| --- | --- | --- |
| 1 | no data | review paper; no original patient data |
| 2 | not dizziness | no reasonable prospect that the study includes data about dizziness or vertigo |
| 3 | not acute | no reasonable prospect that the study includes data about *acute* (<72 hours) dizziness or vertigo |
| 4 | not diagnosis | no reasonable prospect that the study includes data about clinical diagnostic accuracy of spontaneous nystagmus in acute central (specifically stroke) or peripheral (specifically vestibular neuritis) disorders |
| 5 | <5 cases | fewer than 5 subjects (total participants reported, including cases and controls) |
| 6 | abstract only | only abstract available (from poster presentation or talk at conference) |
| 7 | other | any other reason abstract is not included |

## **Full-text review coding rules**

1) Coding status options are “Yes” or “No”.

2) Each "No" should be coded with a reason for exclusion.

3) Reasons for exclusion are listed below 0-6. Go through them in order from 0 to 6 for each full manuscript, coding the first reason for exclusion only, not multiple reasons for exclusion.

4) Two independent raters (AAT/MW) will code reason for exclusion, and we will mandate agreement on exclusion reason at the manuscript level.

5) Coding differences will be adjudicated or consensus will be developed through dialogue.

## **Full-text reasons for exclusion**

| 0 | not English | manuscript is not in English |
| --- | --- | --- |
| 1 | no data | review paper; no original patient data |
| 2 | not dizziness | the study does not include data about dizziness or vertigo |
| 3 | not acute | the study does not include data about *acute* (<72 hours) dizziness or vertigo obtained during the *acute phase* (<72 hours) of disease |
| 4 | not diagnosis | no reasonable prospect that the study includes data about clinical diagnostic accuracy of spontaneous nystagmus in acute central (specifically stroke) or peripheral (specifically vestibular neuritis) disorders |
| 5 | <5 cases | fewer than 5 subjects (total participants reported, including cases and controls) |
| 6 | other | any other reason full text is not included |

## **Search Results**

Our search identified 4186 unique citations, of which 3967 (94.8%) were excluded at the abstract level (see PRISMA flow chart). We did not demand concordance on reason for abstract exclusion, but, among those abstracts with concordant reasons for exclusion (26.4%, n=1106), the distribution was as follows: 26.9% **did not include data about acute (<72h) vertigo or dizziness, 25.6% had no original data, 22.2% were** not **about** clinical diagnostic accuracy**; 20.4% were not about vertigo or dizziness, 4.8% had fewer than 5 subjects studied, and 0.1% were not in English.**

We sought to examine 219 full manuscripts (this included 4 articles identified by hand-search). After initial screening, there were a total of 43 disagreements (19.6%) about study inclusion for the two reviewers (MW and AAT [search period: 1980-2022], kappa=0.44). These differences were resolved by discussion and – if needed - adjudication by a third reviewer. Overall agreement on reason for exclusion was 58.4%. We demanded concordance on reason for full-text exclusion and resolved differences by discussion.

At the end of our full-text review, 180 studies were excluded and 39 were considered eligible (see PRISMA flow chart in main manuscript [Figure 1]). These eligible studies represented 1.0% of the total (n=4186). Among all full-text manuscripts excluded (4.3%), the distribution of reason for exclusion was as follows: **did not include data about acute (<72h) vertigo or dizziness** (54.4%), were **not about** clinical diagnostic accuracy (40.0%), **had fewer than 5 subjects studied** (2.2%), contained no original data (2.2%), and lacking access to the full-text manuscript (1.1%).

For 13 studies we attempted to contact the first or corresponding author for additional study information. Eight authors responded and provided additional information.

**Appendix 2 - QUADAS-2 assessment of included studies**

For included studies, two independent raters (MW/AAT) assessed the risk of bias or applicability concerns using QUADAS-2 (Whiting et al., 2011) tailored study criteria, resolving disagreements by discussion. The QUADAS-2 tool for quality rating of diagnostic accuracy studies consists of four core domains (patient selection, index test, reference standard, and flow and timing) (Whiting et al., 2011). Risk of bias is assessed for all four domains, and applicability is assessed for the first three domains. Thus, seven items per study are assessed to rate quality of evidence (see Table S1). For each item, pre-specified conditions must be met to qualify for “low risk” of bias. The tool’s authors recommend review-specific tailoring of the rating criteria and process (Whiting et al., 2011), which we did for all seven criteria (Box 1).

Overall, very few studies were low risk of bias and low risk of applicability concerns in all items (Kattah et al., 2009b;Newman-Toker et al., 2013;Mantokoudis et al., 2021), whereas most studies (n=32) demonstrated high risk of bias for at least one item. Most frequently, there was a high risk of bias regarding patient selection (n=28 studies).

*eBox 1. Tailored QUADAS-2 rating criteria for key domains of bias or applicability concerns (modified after (Tarnutzer et al., 2023))*

- Patient selection (bias): Studies were rated as *low* risk if the population was well-described and unbiased. Studies were rated as *unclear* if patients were drawn from a prospective patient registry without further explanation of original selection. Studies were rated as *high* risk if patients were chosen in non-consecutive/non-random fashion or if they studied a known clinical or demographic subgroup in whom test properties of bedside findings were likely overstated (e.g., sensitivity of neurologic findings for stroke if only patients with such findings were investigated) or understated (e.g., eye movement exams applied to incorrect subpopulations of dizziness/vertigo, such as head impulse testing applied to patients with episodic, positional vertigo).
- Patient selection (applicability concerns): Studies were rated as *low* risk if they used clear, accepted definitions for AVS and/or positional EVS (eBox 2) (Newman-Toker and Edlow, 2015;Newman-Toker, 2017). Studies were rated as *unclear* if patients were classified but without clear definitions (e.g., said to have AVS, but without defining AVS directly or referencing previously-published criteria). Studies were rated as *high* risk if patients were classified with clear definitions that did not align with accepted definitions.
- Index test (bias): Studies were rated as *low* risk if bedside (index) tests were performed by subspecialists (neuro-ophthalmology, neuro-otology, or other clinicians routinely using vestibular tests [e.g., vestibular physical therapists, vestibular lab technicians]) or trained specialists (e.g., neurologists) or generalists (e.g., ED physicians) where rater skills were shown to be accurate and reliable. Studies were rated *unclear* if tests were performed by untrained specialists (general neurologists or otolaryngologists); specialty trainees (e.g., neurology or otolaryngology residents); if the training of those who performed the bedside examinations was either mixed (e.g., specialists and ED physicians); or if training or masking was not specifically reported. Studies were rated as having *high* risk if more than 20% of tests were performed by generalists who were untrained or whose test performance was of low quality or not assessed.
- Index test (applicability concerns): Studies were rated as *low* risk if bedside test techniques were described in sufficient detail to match accepted standards. Studies were rated as having *high* risk if bedside test techniques were described or demonstrated (e.g., by video) to not match accepted standards or were inconsistently applied. Studies were rated as *unclear* if bedside techniques were not described sufficiently.
- Reference standard (bias): The risk of bias in the reference standard was determined using rules specific to each diagnosis (see eBox 3).
- Reference standard (applicability concerns): Studies were rated as *low* risk if standard diagnostic criteria were used to define stroke and/or peripheral vestibular disorders, and these criteria were compatible with existing consensus definitions and guidelines (Easton et al., 2009) (Fife et al., 2008;Lempert et al., 2012;Lopez-Escamez et al., 2015;von Brevern et al., 2015;Bhattacharyya et al., 2017). Studies were rated as *unclear* if definitions were not reported. Studies were rated as *high* risk if non-standard definitions were applied.
- Flow and timing (bias): Studies were rated as *low* risk if over 80% of patients underwent the same (or an equivalently valid) reference standard in a timely manner. Studies were rated as *unclear* if details were not reported. Studies were rated as *high* risk if the reference standard varied or timing was not appropriate (e.g., if an MRI reference standard was too early or too late, lowering its sensitivity) in more than 20%.

Abbreviations: AVS – acute vestibular syndrome; ED – emergency department; EVS – episodic vestibular syndrome.

*eBox 2. Consensus (ICVD) Definitions for Vestibular Syndromes (i.e., dizziness/vertigo subpopulations)*

- Acute Vestibular Syndrome (AVS): A clinical syndrome of acute-onset, continuous vertigo, dizziness, or unsteadiness lasting days to weeks, and generally including features suggestive of new, ongoing vestibular system dysfunction (e.g., vomiting, nystagmus, postural instability).
- Episodic Vestibular Syndrome (EVS): A clinical syndrome of transient vertigo, dizziness, or unsteadiness lasting seconds to hours, occasionally days, and generally including features suggestive of temporary, short-lived vestibular system dysfunction (e.g., nausea, nystagmus, sudden falls).
- Chronic Vestibular Syndrome (CVS): A clinical syndrome of chronic vertigo, dizziness, or unsteadiness lasting months to years and generally including features suggestive of persistent vestibular system dysfunction (e.g., oscillopsia, nystagmus, gait unsteadiness).

Abbreviations: ICVD – International Classification of Vestibular Disorders

*eBox 3. Valid reference standards for central and peripheral vestibular disorders with risk of bias (modified after (Tarnutzer et al., 2023))*

- Stroke: Strokes must have been confirmed by neuroimaging to be considered valid. The type of imaging and imaging result determined the QUADAS-2 risk of bias on reference standard. To be considered *low* risk, strokes had to be clinically deemed causal and confirmed to be new/acute lesions by neuroimaging. To be considered causal, rather than incidental, strokes must have been found in a brain region appropriate to the clinical syndrome (brainstem, cerebellum, thalamus, or parieto-insular region if hemispheric). To be confirmed new/acute by neuroimaging, strokes must have been demonstrated by (1) MRI-DWI showing restricted diffusion; (2) clearly new lesion evident by post-onset serial CT or MRI (e.g., no lesion on day #1 but present on day #3); or (3) acute brain hemorrhages or large ischemic strokes clearly evident by CT (e.g., based on demarcation, attenuation, and/or mass effect). Studies that demonstrably fell short of these requirements were rated as *high* risk, whereas studies not providing sufficient detail on neuroimaging were considered of *unclear* risk. If testing protocols were non-uniform across patients, no more than 20% of patients could be in a higher risk category to still maintain the lower risk category for QUADAS-2 status.
- Vestibular neuritis (without hearing loss) or labyrinthitis (with hearing loss): Patients must have had an AVS clinical presentation, a spontaneous or gaze-evoked nystagmus consistent with Alexander’s law, evidence of a unilateral vestibular deficit on quantitative physiologic testing (caloric or quantified head impulse testing), and either no associated (new) neurologic deficits to suggest a central nervous system disorder or negative neuroimaging (by CT or MRI) to be considered valid. The type of imaging and time window from onset of AVS symptoms determined the QUADAS-2 risk of bias on reference standard: *low* risk (MRI-DWI 48 hours to 14 days, inclusive), *unclear* risk (MRI-DWI 0-48 hours), *high* risk (otherwise). If the imaging type or time windows were non-uniform across patients, no more than 20% of patients could be in a higher risk category to still maintain the lower risk category for QUADAS-2 status.

Abbreviations: AVS – acute vestibular syndrome; DWI – diffusion weighted imaging; EVS – episodic vestibular syndrome; ICVD – International Classification of Vestibular Disorders

**Table S1 – QUADAS2 risk of bias assessment of all included studies (n=39)**

|  |  | **Risk of Bias** | | | | **Applicability Concerns** | | |
| --- | --- | --- | --- | --- | --- | --- | --- | --- |
| **Citation** | **Sample size (n)** | **Patient Selection** | **Index Test** | **Reference Standard** | **Flow & Timing** | **Patient Selection** | **Index Test** | **Reference Standard** |
| Bassetti et al. (Bassetti et al., 1997) | 6 | High risk | Low risk | Low risk | Low risk | Unclear risk | Unclear risk | Low risk |
| Braun et al. (Braun et al., 2011) | 11 | High risk | Unclear risk | Low risk | Low risk | Unclear risk | Low risk | Low risk |
| Calic et al. (Calic et al., 2020) | 8 | High risk | Low risk | Unclear risk | Low risk | Low risk | Low risk | Low risk |
| Carmona et al. (Carmona et al., 2016) (2016) | 114 | Low risk | Low risk | Unclear risk | Low risk | Low risk | Low risk | Low risk |
| Chase et al. (Chase et al., 2012) | 12 | High risk | Unclear risk | Unclear risk | Unclear risk | Unclear risk | Unclear risk | Low risk |
| Chen et al. (Chen et al., 2011) (2011) | 24 | Low risk | Unclear risk | Low risk | Low risk | Low risk | Low risk | Unclear risk |
| Choi et al. (Choi et al., 2007) | 9 | High risk | Low risk | Low risk | Low risk | Unclear risk | Low risk | Low risk |
| Choi et al. (Choi et al., 2018) | 35 | High risk | Low risk | Low risk | Low risk | Low risk | Unclear risk | Low risk |
| Choi et al. (Choi et al., 2015) | 8 | High risk | Low risk | Low risk | Low risk | Low risk | Low risk | Low risk |
| Dieterich et al. (Dieterich et al., 2018) | 23 | High risk | Low risk | Low risk | Low risk | Unclear risk | Unclear risk | Low risk |
| Eggers et al. (Eggers et al., 2009) | 5 | High risk | Unclear risk | Low risk | Low risk | Low risk | Unclear risk | Low risk |
| Guler et al. (Guler et al., 2017) | 40 | Low risk | Unclear risk | Unclear risk | Low risk | Low risk | Unclear risk | Low risk |
| Helmchen et al. (Helmchen et al., 2002) | 11 | High risk | Low risk | Low risk | Low risk | Unclear risk | Low risk | Low risk |
| Honda et al. (Honda et al., 2014) | 41 | Low risk | Unclear risk | Unclear risk | Low risk | Low risk | Unclear risk | Low risk |
| Kattah et al. (2009)(Kattah et al., 2009b) | 101 | Low risk | Low risk | Low risk | Low risk | Low risk | Low risk | Low risk |
| Kim et al. (Kim et al., 2016) | 10 | High risk | Unclear risk | High risk | Unclear risk | Unclear risk | Low risk | Low risk |
| Kim and Heo (Kim and Heo, 1996) | 30 | High risk | Unclear risk | Low risk | Low risk | Unclear risk | Unclear risk | Low risk |
| Kim et al. (Kim et al., 2017) | 201 | High risk | Unclear risk | Unclear risk | Unclear risk | Low risk | Unclear risk | Unclear risk |
| Kim et al. (Kim and Han, 2009) | 86 | High risk | Unclear risk | Low risk | Low risk | High risk | Low risk | Low risk |
| Kim and Kim (Kim and Kim, 2012) | 9 | High risk | Low risk | Unclear risk | Low risk | Low risk | Low risk | Low risk |
| Lee et al. (Lee et al., 2002) | 12 | High risk | Low risk | Low risk | Low risk | Unclear risk | Low risk | Low risk |
| Lee et al. (Lee et al., 2006) | 25 | High risk | Low risk | Low risk | Low risk | Low risk | Low risk | Low risk |
| Lee et al. (Lee et al., 2008) | 12 | High risk | Low risk | Low risk | Low risk | Unclear risk | Low risk | Low risk |
| Lee et al. (Lee et al., 2019a) | 30 | High risk | Low risk | Low risk | Low risk | Low risk | Low risk | Low risk |
| Lee et al. (Lee et al., 2019b) | 133 | High risk | Low risk | High risk | Unclear risk | Low risk | Low risk | Unclear risk |
| Lee et al. (Lee et al., 2018) | 31 | High risk | Unclear risk | Unclear risk | Unclear risk | Low risk | Unclear risk | Low risk |
| Lee et al. (Lee et al., 2015) | 18 | High risk | Low risk | Low risk | Low risk | Low risk | Low risk | Low risk |
| Ling et al. (Ling et al., 2019) | 69 | High risk | Low risk | Unclear risk | Low risk | Low risk | Low risk | Low risk |
| Mantokoudis et al. (Mantokoudis et al., 2021) | 63 | Low risk | Low risk | Low risk | Low risk | Low risk | Low risk | Low risk |
| Morita et al. (Morita et al., 2011) | 8 | High risk | Unclear risk | Low risk | Low risk | Unclear risk | Low risk | Low risk |
| Newman-Toker et al. (Newman-Toker et al., 2013) | 191 | Low risk | Low risk | Low risk | Low risk | Low risk | Low risk | Low risk |
| Nham et al. (Nham et al., 2023) | 205 | Low risk | Low risk | High risk | Low risk | Low risk | Low risk | Low risk |
| Nham et al. (Nham et al., 2022) | 97 | Low risk | Low risk | High risk | Low risk | Low risk | Low risk | Low risk |
| Norrving et al. (Norrving et al., 1995) | 24 | Low risk | Low risk | High risk | High risk | Low risk | Unclear risk | Low risk |
| Ogawa et al. (Ogawa et al., 2013) | 18 | High risk | Unclear risk | Low risk | Low risk | Unclear risk | Unclear risk | Low risk |
| Ogawa et al.(Ogawa et al., 2017) | 7 | High risk | Unclear risk | Low risk | Low risk | Unclear risk | Unclear risk | Low risk |
| Ogawa et al. (Ogawa et al., 2016) | 10 | High risk | Unclear risk | Low risk | Low risk | Unclear risk | Low risk | Low risk |
| Pavlin-Premrl et al. (Pavlin-Premrl et al., 2015) | 25 | Low risk | Low risk | High risk | High risk | High risk | Low risk | Low risk |
| Weng et al. (Weng and Young, 2014) | 14 | High risk | Unclear risk | Low risk | Low risk | Low risk | Low risk | Low risk |

# **Appendix 3 – additional tables**

## **Table S2: Studies included in the systematic review**

| **Author (citation, year)** | **Total Dizziness Sample (studied, % females)** | **Definition of total sample / dizzy sample** | **Data collection (source, analysis)** | **Setting (country)** | **Bedside testing performed by** | **Vertical / torsional eye movements assessed** | **Testing performed with fixation preserved or suppressed?** | **Mean age (standard deviation, range) [years]** | **Special comments** |
| --- | --- | --- | --- | --- | --- | --- | --- | --- | --- |
| Bassetti et al. (Bassetti et al., 1997) | NR (6, 50%) | Patients with medial medullary stroke | Retrospective (hospital admission population, cross-sectional) | Two academic hospitals (Switzerland) | Board-certified stroke neurologists | Yes | NR | 41.8 (14.9, 30-58) | Clinical examination was performed within 1 week after symptom onset only. While no further details on timing were provided, it is reasonable to assume that initial examination was done acutely (i.e., within < 24 hours after symptom onset) considering the obvious focal neurologic symptoms including acute hemiparesis in all patients. |
| Braun et al. (Braun et al., 2011) | NR (11, 36%) | Patients with vertigo and initially missed stroke | Retrospective (hospital admission population, cross-sectional) | Single academic hospital (Austria) | ENT specialists | Yes | NR | 62.5 (14.7, 27-76) | Central nystagmus patterns were exclusion criterion. 1 patient with a sinus vein thrombosis was excluded as no stroke was reported. |
| Calic et al. (Calic et al., 2020) | 5305 (8, 50%) | Patients with vestibular migraine presenting as acute peripheral vestibulopathy | Retrospective (Neuro-otology clinic population, cross-sectional) | Single academic hospital (Australia) | Experienced neuro-otologists | Yes | NR | 46.9 (14.2, 35-76) | Central nystagmus patterns were exclusion criterion. Two patients were excluded because of delay to testing (>3 days) or unknown timing. |
| Carmona et al. (Carmona et al., 2016) (2016) | 1218 (114, 58%) | ED presentation due to “dizziness” | Retrospective (ED population, cross-sectional) | Single academic hospital (Argentina) | Trained neurology residents supervised by experienced neuro-otologists | Yes | Fixation suppressed | cAVS: 57·9 (11, NR)  pAVS: 43·3 (14·9, NR) |  |
| Chase et al. (Chase et al., 2012) | 325 (12, 42%) | Patients presenting to ED with vertigo that had confirmed stroke on MRI | Retrospective (ED population, cross-sectional) | Single academic hospital (USA) | Neurology consult team | Yes | NR | 61.5 (16.3, 25-89) |  |
| Chen et al. (Chen et al., 2011) (2011) | 36 (24, 37%) | ED presentation with „acute prolonged rotatory vertigo associated with nausea and/or vomiting, without other brainstem signs“ | Prospective (ED population, cross-sectional) | Single academic hospital (Australia) | Neurologists with 4 hours of training in neuro-otology | Yes | Fixation preserved | 64.0 (13, 42-83) |  |
| Choi et al. (Choi et al., 2007) | NR (9, 55%) | Patients with acute lateral medullary infarction | Prospective (ED population, cross-sectional) | Single academic hospital (South Korea) | Experienced neuro-otologists | Yes | Both with fixation preserved and suppressed | 58.6 (9.8, | Only those 9 patients with evaluation in the acute stage (i.e., within 72h) were included, seven patients with delayed testing were excluded |
| Choi et al. (Choi et al., 2018) | 1846 (35, 26%) | Stroke patients with initially MRI-DWI negative PCS and acute vertigo | Retrospective analysis of a prospective registry (ED population, cross-sectional) | Single academic hospital (South Korea) | Experienced neuro-otologists | Yes | NR | 63 (13, 31-89) |  |
| Choi et al. (Choi et al., 2015) | NR (9, 63%) | Stroke patients with isolated unilateral inferior cerebellar peduncle lesions | Prospective (ED population, cross-sectional) | Single academic hospital (South Korea) | Experienced neuro-otologists | Yes | Both with fixation preserved and suppressed | 53.6 (21.4, 18-83) | No bedside assessment of spontaneous nystagmus, only quantitative testing reported. |
| Dieterich et al. (Dieterich et al., 2018) | 158 (23, 64%) | Patients with acute vestibular and/or ocular motor symptoms due to unilateral midbrain syndromes | Retrospective (ED population, cross-sectional) | Single academic hospital (Germany) | Experienced neuro-otologists | Yes | NR | 56.1 (18.0, 26-82) | Only those 23 out of 63 midbrain strokes that reported vertigo or dizziness were included. |
| Eggers et al. (Eggers et al., 2009) | NR (5, 40%) | Patients with medulla oblongata syndrome, sx-onset <24h and ipsilateral axial lateropulsion | Retrospective (ED population, cross-sectional) | Single academic hospital (Germany) | Neurologists, no further information provided about sub-specialization | Yes | NR | 69 (10, 54-82) | Only 5 out of 13 patients reported dizziness, only those were included |
| Guler et al. (Guler et al., 2017) | NR (52, 27%) | Patients presenting with acute vestibular syndrome | Prospective (ED population, cross-sectional) | Single academic hospital (Turkey) | Both ED specialists and experienced neuro-otologists | No | NR | cAVS: 52 (NR, NR)  pAVS: 46 (, NR) | No reporting on torsional and/or vertical SN |
| Helmchen et al. (Helmchen et al., 2002) | NR (11, 45%) | Patients with acute vertigo, torsional nystagmus and mesencephalic lesions | Prospective (ED population, cross-sectional) | Single academic hospital (Germany) | Experienced neuro-otologists | Yes | Both with fixation preserved and suppressed | 59.6 (13.4, | Torsional SN was an inclusion criterion |
| Honda et al. (Honda et al., 2014) | 358 (41, 41%) | Patients with acute imbalance/vertigo without obvious neurologic signs and no abnormalities on CT | Prospective (ED population, cross-sectional) | Single academic hospital (Japan) | Neurologists, no further information provided about sub-specialization | Yes | NR | 70.2 (11.2, NR) | 1 patient with a transient-ischemic attack is included |
| Kim et al. (Kim et al., 2016) | NR (10, 90%) | Patients with serous labyrinthitis | Likely prospective (ED population, cross-sectional) | Single academic hospital (South Korea) | ENT specialists | No | NR | 54.8 (11.9, 36-75) | MRI of the brain performed in only 6/13 patients. No reporting on torsional and/or vertical SN. Note that 3/13 patients were removed as testing was not done within 72 hours. |
| Kim and Heo (Kim and Heo, 1996) | 154 (30, 43%) | Patients with acute vertigo and confirmed stroke (CT or MRI) | Prospective (ED population, cross-sectional) | Single academic hospital (South Korea) | Neurologists, no further information provided about sub-specialization | No | NR | 59.1 (NR, NR) | No reporting on torsional and/or vertical SN |
| Kim et al. (Kim et al., 2017) | NR (201, 42%) | Patients with vestibular neuritis and no hearing loss, no MRI abnormalities | Retrospective (ED population, cross-sectional) | Single academic hospital (South Korea) | ENT specialists | No | NR | 55 (10, 23-77) | No reporting on torsional and/or vertical SN |
| Kim et al. (Kim and Han, 2009) | 100 (86, 26%) | Patients with isolated medial medullary infarction admitted with 7 days of sx-onset and without previous strokes | Prospective (ED population, cross-sectional) | Single academic hospital (South Korea) | Neurologists, no further information provided about sub-specialization | Yes | NR | 62 (10, NR) | Only a subset of patients (n=51) reported acute vertigo/dizziness. Nystagmus patterns, however, were not provided separately for those with vertigo/dizziness, thus some of the patients included may not have presented with vertigo/dizziness. Patients were included up to 7 days after symptom onset. With focal neurologic signs including motor dysfunction in 91%, however, early ED presentation in most patients is likely. |
| Kim and Kim (Kim and Kim, 2012) | 703 (9, 67%) | Patients with inferior vestibular neuritis | Retrospective (ED population, cross-sectional) | Single academic hospital (South Korea) | Experienced neuro-otologists | Yes | Both with fixation preserved and suppressed | 51.9 (19, 15-75) |  |
| Lee et al. (Lee et al., 2002) | NR (12, 63%) | Patients with MRI-confirmed AICA stroke | Prospective (ED population, cross-sectional) | Single academic hospital (South Korea) | Experienced neuro-otologists | Yes | NR | 60.6 (13.5, 28-83) |  |
| Lee et al. (Lee et al., 2006) | 240 (25, 56%) | Patients with pseudo vestibular neuritis and isolated cerebellar infarction | Retrospective (ED population, cross-sectional) | Single academic hospital (South Korea) | Experienced neuro-otologists | No | NR | 63.7 (10.6, 37-80) | No reporting on torsional and/or vertical SN |
| Lee et al. (Lee et al., 2008) | NR (12, 33%) | Patients with AICA stroke | Retrospective (ED population, cross-sectional) | Single academic hospital (South Korea) | Experienced neuro-otologists | Yes | NR | 61.2 (12.4, 35-84) |  |
| Lee et al. (Lee et al., 2019a) | 412 (30, NR) | Patients with acute vertigo and spontaneous nystagmus, but normal vHIT | Retrospective (ED population, cross-sectional) | Single academic hospital (South Korea) | ENT specialists | No | Fixation preserved | 48.3 (14.3, 19-79) | Horizontal SN was inclusion criterion, whereas presence of vertical / torsional nystagmus was an exclusion criterion. Note that SN characteristics were reported only with sufficient detail for 17 patients with Menière’s disease. Thus, remaining peripheral (n=8) and central (n=5) AVS patients were excluded from the analysis. |
| Lee et al. (Lee et al., 2019b) | NR (133, 47%) | Patients with acute vestibular neuritis | Retrospective (ED population, cross-sectional) | Single academic hospital (South Korea) | ENT specialists | Yes | NR | 54.4 (8.1, 20-80) | Horizontal spontaneous nystagmus was inclusion criterion |
| Lee et al. (Lee et al., 2018) | NR (31, 65%) | Patients with isolated audiovestibular loss and SN without brainstem lesions | Retrospective (ED population, cross-sectional) | Single academic hospital (South Korea) | ENT specialists | No | NR | cAVS: 65.5 (2.1, NR)  pAVS: 51.2 (17.2, NR) | Patients had no vertical nystagmus. No reporting on presence/absence of torsional nystagmus. Note that patients with symptoms less than 1 week were included and no further details about timing are provided. |
| Lee et al. (Lee et al., 2015) | 172 (18, 22%) | Patients with infarction of the dorsal medulla | Retrospective (ED population, cross-sectional) | Single academic hospital (South Korea) | Experienced neuro-otologists | Yes | Both with fixation preserved and suppressed | 61.1 (11.8, 33-73) | While patients were included up to 10 days after symptom onset, clinical oculomotor testing was performed within 72 hours in >75%. |
| Ling et al. (Ling et al., 2019) | NR (69, 33%) | Patients with posterior cerebellar infarction or unilateral VN | Retrospective (ED population, cross-sectional) | Single academic hospital (China) | Not reported | Yes | Both with fixation preserved and suppressed | cAVS: 62.0 (12.6, 42-84)  pAVS: 55.9 (15.7, NR) | For the VN cases, presence of spontaneous horizontal or horizontal/torsional nystagmus was mandatory. Spontaneous nystagmus beating patterns are reported both in a qualitative and quantitative way, thus potentially no bedside examination was performed. |
| Mantokoudis et al. (Mantokoudis et al., 2021) | 1646 (63, 57%) | Patients with acute, persistent dizziness fulfilling all criteria for an AVS | Prospective (ED population, cross-sectional) | Single academic hospital (Switzerland) | Experienced neuro-otologists | Yes | Both with fixation preserved and suppressed | cAVS: 63.8 (13.4, NR)  pAVS: 53.4 (16.2, NR) | Spontaneous horizontal nystagmus was inclusion criterion |
| Morita et al. (Morita et al., 2011) | NR (8, 63%) | Isolated cAVS with initially negative MRI-DWI | Retrospective (ED population, cross-sectional) | Single academic hospital (Japan) | Not reported | Yes | NR | 71.5 (7.3, 61-84) |  |
| Newman-Toker et al. (Newman-Toker et al., 2013)  Preliminary report in Kattah et al. (Kattah et al., 2009a) | 191 (191, 40%) | “Patients with at least 1 hour of acute, persistent, continuous vertigo or dizziness with spontaneous or gaze-evoked nystagmus, plus nausea or vomiting, head motion intolerance, and new gait unsteadiness (i.e., AVS), presenting within 1 week of symptom onset“ | Prospective (ED / hospital admission population, cross-sectional) | Single academic hospital (USA) | Experienced neuro-otologists | Yes | Fixation preserved (and with fixation suppressed in some) | 61.0 (NR, 18-92) | Patients with initial negative MRI-DWI underwent repeat MRI-DWI for unexplained signs suggesting brainstem location. Based on the dataset provided by the corresponding author, we included one additional patient that was not considered in the original publication. Most of the patients were examined within 24 hours of symptom onset and almost all within 72 hours. |
| Nham et al. (Nham et al., 2023) | NR (205, 38%) | Patients with either radiologically confirmed posterior circulation stroke with vertigo/dizziness or patients with acute VN presenting as AVS. | Prospective (ED population, cross-sectional) | Single academic hospital (Australia) | Experienced neuro-otologists | Yes | Fixation suppressed (and with fixation preserved in some) | cAVS: 67.0 (12.3, NR)  pAVS: 56.9 (17.0, NR) | SN was inclusion criterion for pAVS, but not for cAVS. Significant overlap with Nham et al. (Nham et al., 2022). Note that only quantitative, bedside video-nystagmography based SN patterns are reported in both studies from Nham et al. Thus, these studies were excluded from the meta-analysis. |
| Norrving et al. (Norrving et al., 1995) | NR (24, 58%) | Patients with acute onset of isolated vertigo lasting > 48 h and no abnormality on neurological examination other than nystagmus. | Prospective (ED population, cross-sectional) | Single academic hospital (Sweden) | Experienced neuro-otologists | Yes | NR | 62.2 (NR, NR) | Only patients aged 50-75 years were included. |
| Ogawa et al. (Ogawa et al., 2013) | NR (18, 50%) | Patients reporting vertigo and / or dizziness with conformed PICA strokes | Retrospective (hospital admission population, cross-sectional) | Single academic hospital (Japan) | Experienced neuro-otologists | No | NR | 65.8 (7.8, 54-81) | No information on whether torsional and / or vertical nystagmus was assessed or not. Note that no information about timing is provided, however, with acute focal neurologic deficits including disturbed stance and gait early presentation to the ED is very likely. |
| Ogawa et al.(Ogawa et al., 2017) | NR (7, 14%) | Patients reporting vertigo and / or dizziness with conformed AICA strokes | Retrospective (hospital admission population, cross-sectional) | Single academic hospital (Japan) | Experienced neuro-otologists | No | NR | 55.1 (13.2, 32-72) | No information on whether torsional and / or vertical nystagmus was assessed or not. Note that no information about timing is provided, however, with acute focal neurologic deficits including disturbed stance and gait early presentation to the ED is very likely. |
| Ogawa et al. (Ogawa et al., 2016) | NR (10, 20%) | Patients with acute vertigo first considered of peripheral origin | Retrospective (hospital admission population, cross-sectional) | Single academic hospital (Japan) | Experienced neuro-otologists | Yes | NR | 65.1 (13.1, 35-83) | Note that one additional patient that was not assessed within 72 hours after symptom onset was not included. |
| Pavlin-Premrl et al. (Pavlin-Premrl et al., 2015) | NR (25, NR) | Patients with acute vertigo brought to the ED | Prospective (ED population, cross-sectional) | Single academic hospital (Australia) | Experienced neuro-otologists | Yes | Fixation preserved (and with fixation suppressed in 11) | 62.7 (NR, 38-91) | Three patients with unclear diagnosis were excluded |
| Weng et al. (Weng and Young, 2014) | 14541 (14, 43%) | Patients with acute vertigo and confirmed PICA/AICA stroke on MRI | Retrospective (ED population, cross-sectional) | Single academic hospital (Taiwan) | ENT specialists | Yes | NR | 56.9 (16.8, |  |

* Level of evidence was determined as defined by (criteria may be found in Appendix 1).

Abbreviations: AICA = anterior inferior cerebellar artery; AVS = acute vestibular syndrome (vertigo, nystagmus, nausea/vomiting, head-motion intolerance, unsteady gait lasting ≥24h); cAVS = central AVS; CT = computer tomography; DWI = diffusion-weighted imaging; ENT = ear-nose-throat; MRI = magnetic resonance imaging; ED = emergency department; NR = not reported; pAVS = peripheral AVS; PICA = posterior inferior cerebellar artery; SN = spontaneous nystagmus; VEMPs = vestibular-evoked myogenic potentials; VN = vestibular neuritis

**Table S3: cAVS – spontaneous nystagmus patterns based on the vascular territory / anatomical region affected**

|  | **nystagmus pattern observed** | | | | | | | | | | |
| --- | --- | --- | --- | --- | --- | --- | --- | --- | --- | --- | --- |
|  | **horizontal only** | **horizontal torsional** | **horizontal vertical** | **horizontal-vertical-torsional** | **torsional vertical** | **torsional only** | **DBN only** | **UBN only** | **vertical only (direction NR)** | **any SN** | **% with SN** |
| **Specific vascular territories** |  |  |  |  |  |  |  |  |  |  |  |
| PICA | 77 | 1 | 7 | 0 | 0 | 0 | 1 | 3 | 0 | 89 | 65.0 |
| AICA | 20 | 20 | 0 | 0 | 0 | 0 | 1 | 0 | 0 | 41 | 82.0 |
| SCA | 5 | 0 | 0 | 0 | 0 | 0 | 0 | 0 | 0 | 5 | 62.5 |
| PICA-AICA | 1 | 0 | 0 | 0 | 0 | 0 | 0 | 0 | 0 | 1 | 33.3 |
| PICA-SCA | 3 | 0 | 0 | 0 | 0 | 0 | 0 | 0 | 0 | 3 | 75.0 |
| **Anatomical description only** |  |  |  |  |  |  |  |  |  |  |  |
| Mesencephalon | 1 | 0 | 1 | 0 | 0 | 3 | 0 | 2 | 0 | 7 | 20.0 |
| Pons | 3 | 0 | 0 | 0 | 0 | 1 | 1 | 0 | 0 | 5 | 38.5 |
| Medulla oblongata |  |  |  |  |  |  |  |  |  |  |  |
| Medial medulla | 32 | 0 | 4 | 0 | 0 | 0 | 1 | 6 | 0 | 43 | 44.8 |
| (dorso-)lateral medulla | 9 | 3 | 1 | 10 | 2 | 0 | 0 | 0 | 0 | 25 | 80.6 |
| Not further specified | 3 | 0 | 0 | 0 | 0 | 1 | 0 | 0 | 0 | 4 | 57.1 |
| Brainstem and / or cerebellum | 35 | 5 | 0 | 0 | 3 | 7 | 2 | 6 | 1 | 59 | 41.0 |
| **Other causes*** | 12 | 0 | 3 | 0 | 1 | 0 | 2 | 1 | 0 | 19 | 70.4 |
| **Total** | 201 | 29 | 16 | 10 | 6 | 12 | 8 | 18 | 1 | 301 | 54.2 |

* Other causes are multiple sclerosis (n=6), supratentorial strokes (n=8), hemorrhages (n=3), paraneoplastic syndromes (n=1), carbamazepine intoxication (n=1)

**References**

Bassetti, C., Bogousslavsky, J., Mattle, H., and Bernasconi, A. (1997). Medial medullary stroke: report of seven patients and review of the literature. *Neurology* 48**,** 882-890.

Bhattacharyya, N., Gubbels, S.P., Schwartz, S.R., Edlow, J.A., El-Kashlan, H., Fife, T., Holmberg, J.M., Mahoney, K., Hollingsworth, D.B., Roberts, R., Seidman, M.D., Steiner, R.W., Do, B.T., Voelker, C.C., Waguespack, R.W., and Corrigan, M.D. (2017). Clinical Practice Guideline: Benign Paroxysmal Positional Vertigo (Update). *Otolaryngol Head Neck Surg* 156**,** S1-S47.

Braun, E.M., Tomazic, P.V., Ropposch, T., Nemetz, U., Lackner, A., and Walch, C. (2011). Misdiagnosis of acute peripheral vestibulopathy in central nervous ischemic infarction. *Otol Neurotol* 32**,** 1518-1521.

Calic, Z., Nham, B., Taylor, R.L., Young, A.S., Bradshaw, A.P., Mcgarvie, L.M., Colebatch, J.G., Cordato, D., Cappelen-Smith, C., and Welgampola, M.S. (2020). Vestibular migraine presenting with acute peripheral vestibulopathy: Clinical, oculographic and vestibular test profiles. *Cephalalgia Reports* 3**,** 2515816320958175.

Carmona, S., Martinez, C., Zalazar, G., Moro, M., Batuecas-Caletrio, A., Luis, L., and Gordon, C. (2016). The Diagnostic Accuracy of Truncal Ataxia and HINTS as Cardinal Signs for Acute Vestibular Syndrome. *Front Neurol* 7**,** 125.

Chase, M., Joyce, N.R., Carney, E., Salciccioli, J.D., Vinton, D., Donnino, M.W., and Edlow, J.A. (2012). ED patients with vertigo: can we identify clinical factors associated with acute stroke? *Am J Emerg Med* 30**,** 587-591.

Chen, L., Lee, W., Chambers, B.R., and Dewey, H.M. (2011). Diagnostic accuracy of acute vestibular syndrome at the bedside in a stroke unit. *J Neurol* 258**,** 855-861.

Choi, J.H., Oh, E.H., Park, M.G., Baik, S.K., Cho, H.J., Choi, S.Y., Lee, T.H., Kim, J.S., and Choi, K.D. (2018). Early MRI-negative posterior circulation stroke presenting as acute dizziness. *J Neurol* 265**,** 2993-3000.

Choi, J.H., Seo, J.D., Choi, Y.R., Kim, M.J., Kim, H.J., Kim, J.S., and Choi, K.D. (2015). Inferior cerebellar peduncular lesion causes a distinct vestibular syndrome. *Eur J Neurol* 22**,** 1062-1067.

Choi, K.D., Oh, S.Y., Park, S.H., Kim, J.H., Koo, J.W., and Kim, J.S. (2007). Head-shaking nystagmus in lateral medullary infarction: patterns and possible mechanisms. *Neurology* 68**,** 1337-1344.

Cohen, J. (1960). A coefficient for agreement for nominal scales. *Educ Psychol Meas* 20**,** 37-46.

Dieterich, M., Glasauer, S., and Brandt, T. (2018). Why acute unilateral vestibular midbrain lesions rarely manifest with rotational vertigo: a clinical and modelling approach to head direction cell function. *J Neurol* 265**,** 1184-1198.

Easton, J.D., Saver, J.L., Albers, G.W., Alberts, M.J., Chaturvedi, S., Feldmann, E., Hatsukami, T.S., Higashida, R.T., Johnston, S.C., Kidwell, C.S., Lutsep, H.L., Miller, E., Sacco, R.L., American Heart, A., American Stroke Association Stroke, C., Council on Cardiovascular, S., Anesthesia, Council on Cardiovascular, R., Intervention, Council on Cardiovascular, N., and Interdisciplinary Council on Peripheral Vascular, D. (2009). Definition and evaluation of transient ischemic attack: a scientific statement for healthcare professionals from the American Heart Association/American Stroke Association Stroke Council; Council on Cardiovascular Surgery and Anesthesia; Council on Cardiovascular Radiology and Intervention; Council on Cardiovascular Nursing; and the Interdisciplinary Council on Peripheral Vascular Disease. The American Academy of Neurology affirms the value of this statement as an educational tool for neurologists. *Stroke* 40**,** 2276-2293.

Eggers, C., Fink, G.R., Moller-Hartmann, W., and Nowak, D.A. (2009). Correlation of anatomy and function in medulla oblongata infarction. *Eur J Neurol* 16**,** 201-204.

Fife, T.D., Iverson, D.J., Lempert, T., Furman, J.M., Baloh, R.W., Tusa, R.J., Hain, T.C., Herdman, S., Morrow, M.J., Gronseth, G.S., and Quality Standards Subcommittee, A.a.O.N. (2008). Practice parameter: therapies for benign paroxysmal positional vertigo (an evidence-based review): report of the Quality Standards Subcommittee of the American Academy of Neurology. *Neurology* 70**,** 2067-2074.

Guler, A., Karbek Akarca, F., Eraslan, C., Tarhan, C., Bilgen, C., Kirazli, T., and Celebisoy, N. (2017). Clinical and video head impulse test in the diagnosis of posterior circulation stroke presenting as acute vestibular syndrome in the emergency department. *J Vestib Res* 27**,** 233-242.

Helmchen, C., Rambold, H., Kempermann, U., Buttner-Ennever, J.A., and Buttner, U. (2002). Localizing value of torsional nystagmus in small midbrain lesions. *Neurology* 59**,** 1956-1964.

Honda, S., Inatomi, Y., Yonehara, T., Hashimoto, Y., Hirano, T., Ando, Y., and Uchino, M. (2014). Discrimination of acute ischemic stroke from nonischemic vertigo in patients presenting with only imbalance. *J Stroke Cerebrovasc Dis* 23**,** 888-895.

Kattah, J.C., Talkad, A.V., Wang, D.Z., Hsieh, Y.H., and Newman-Toker, D.E. (2009a). HINTS to diagnose stroke in the acute vestibular syndrome: three-step bedside oculomotor examination more sensitive than early MRI diffusion-weighted imaging. *Stroke.* 40**,** 3504-3510.

Kattah, J.C., Talkad, A.V., Wang, D.Z., Hsieh, Y.H., and Newman-Toker, D.E. (2009b). HINTS to diagnose stroke in the acute vestibular syndrome: three-step bedside oculomotor examination more sensitive than early MRI diffusion-weighted imaging. *Stroke* 40**,** 3504-3510.

Kim, C.H., Yang, Y.S., Im, D., and Shin, J.E. (2016). Nystagmus in patients with unilateral acute otitis media complicated by serous labyrinthitis. *Acta Otolaryngol* 136**,** 559-563.

Kim, G.W., and Heo, J.H. (1996). Vertigo of cerebrovascular origin proven by CT scan or MRI: pitfalls in clinical differentiation from vertigo of aural origin. *Yonsei Med J* 37**,** 47-51.

Kim, H.J., Kim, D.Y., Hwang, J.H., and Kim, K.S. (2017). Vestibular Neuritis With Minimal Canal Paresis: Characteristics and Clinical Implication. *Clin Exp Otorhinolaryngol* 10**,** 148-152.

Kim, J.S., and Han, Y.S. (2009). Medial medullary infarction: clinical, imaging, and outcome study in 86 consecutive patients. *Stroke* 40**,** 3221-3225.

Kim, J.S., and Kim, H.J. (2012). Inferior vestibular neuritis. *J Neurol* 259**,** 1553-1560.

Lee, H., Sohn, S.I., Cho, Y.W., Lee, S.R., Ahn, B.H., Park, B.R., and Baloh, R.W. (2006). Cerebellar infarction presenting isolated vertigo: frequency and vascular topographical patterns. *Neurology* 67**,** 1178-1183.

Lee, H., Sohn, S.I., Jung, D.K., Cho, Y.W., Lim, J.G., Yi, S.D., Lee, S.R., Sohn, C.H., and Baloh, R.W. (2002). Sudden deafness and anterior inferior cerebellar artery infarction. *Stroke* 33**,** 2807-2812.

Lee, H., Yi, H.A., Lee, S.R., Lee, S.Y., and Park, B.R. (2008). Ocular torsion associated with infarction in the territory of the anterior inferior cerebellar artery: frequency, pattern, and a major determinant. *J Neurol Sci* 269**,** 18-23.

Lee, J.Y., Kim, C.H., Park, J.S., and Kim, M.B. (2019a). Peripheral Vestibulopathy Presenting as Acute Vertigo and Spontaneous Nystagmus with Negative Video Head Impulse Test. *Otolaryngol Head Neck Surg* 160**,** 894-901.

Lee, J.Y., Park, J.S., and Kim, M.B. (2019b). Clinical Characteristics of Acute Vestibular Neuritis According to Involvement Site. *Otol Neurotol* 40**,** 797-805.

Lee, S.J., Lee, S.A., Kim, B.G., Hong, H.S., Lee, J.Y., and Lee, J.D. (2018). Feasibility of magnetic resonance imaging in the differential diagnosis of isolated acute audiovestibular loss. *J Vestib Res* 28**,** 385-391.

Lee, S.U., Park, S.H., Park, J.J., Kim, H.J., Han, M.K., Bae, H.J., and Kim, J.S. (2015). Dorsal Medullary Infarction: Distinct Syndrome of Isolated Central Vestibulopathy. *Stroke* 46**,** 3081-3087.

Lempert, T., Olesen, J., Furman, J., Waterston, J., Seemungal, B., Carey, J., Bisdorff, A., Versino, M., Evers, S., and Newman-Toker, D. (2012). Vestibular migraine: diagnostic criteria. *J Vestib Res* 22**,** 167-172.

Ling, X., Sang, W., Shen, B., Li, K., Si, L., and Yang, X. (2019). Diagnostic value of eye movement and vestibular function tests in patients with posterior circulation infarction. *Acta Otolaryngol* 139**,** 135-145.

Lopez-Escamez, J.A., Carey, J., Chung, W.H., Goebel, J.A., Magnusson, M., Mandala, M., Newman-Toker, D.E., Strupp, M., Suzuki, M., Trabalzini, F., Bisdorff, A., Classification Committee of the Barany, S., Japan Society for Equilibrium, R., European Academy Of, O., Neurotology, Equilibrium Committee of the American Academy Of, O.-H., Neck, S., and Korean Balance, S. (2015). Diagnostic criteria for Meniere's disease. *J Vestib Res* 25**,** 1-7.

Mantokoudis, G., Wyss, T., Zamaro, E., Korda, A., Wagner, F., Sauter, T.C., Kerkeni, H., Kalla, R., Morrison, M., and Caversaccio, M.D. (2021). Stroke Prediction Based on the Spontaneous Nystagmus Suppression Test in Dizzy Patients: A Diagnostic Accuracy Study. *Neurology* 97**,** e42-e51.

Morita, S., Suzuki, M., and Iizuka, K. (2011). False-negative diffusion-weighted MRI in acute cerebellar stroke. *Auris Nasus Larynx* 38**,** 577-582.

Newman-Toker, D. (Year). "Vestibular syndrome definitions for the International Classification of Vestibular Disorders", in: *Bárány Classification Committee Meeting on March 10-11, 2017*).

Newman-Toker, D.E., and Edlow, J.A. (2015). TiTrATE: A Novel, Evidence-Based Approach to Diagnosing Acute Dizziness and Vertigo. *Neurol Clin* 33**,** 577-599, viii.

Newman-Toker, D.E., Kerber, K.A., Hsieh, Y.H., Pula, J.H., Omron, R., Saber Tehrani, A.S., Mantokoudis, G., Hanley, D.F., Zee, D.S., and Kattah, J.C. (2013). HINTS outperforms ABCD2 to screen for stroke in acute continuous vertigo and dizziness. *Acad Emerg Med* 20**,** 986-996.

Nham, B., Akdal, G., Young, A.S., Ozcelik, P., Tanriverdizade, T., Ala, R.T., Bradshaw, A.P., Wang, C., Men, S., Giarola, B.F., Black, D.A., Thompson, E.O., Halmagyi, G.M., and Welgampola, M.S. (2023). Capturing nystagmus in the emergency room: posterior circulation stroke versus acute vestibular neuritis. *J Neurol* 270**,** 632-641.

Nham, B., Reid, N., Bein, K., Bradshaw, A.P., Mcgarvie, L.A., Argaet, E.C., Young, A.S., Watson, S.R., Halmagyi, G.M., Black, D.A., and Welgampola, M.S. (2022). Capturing vertigo in the emergency room: three tools to double the rate of diagnosis. *J Neurol* 269**,** 294-306.

Norrving, B., Magnusson, M., and Holtas, S. (1995). Isolated acute vertigo in the elderly; vestibular or vascular disease? *Acta Neurol Scand* 91**,** 43-48.

Ogawa, K., Suzuki, Y., Oishi, M., Kamei, S., Shigihara, S., and Nomura, Y. (2013). Clinical study of medial area infarction in the region of posterior inferior cerebellar artery. *J Stroke Cerebrovasc Dis* 22**,** 508-513.

Ogawa, K., Suzuki, Y., Takahashi, K., Akimoto, T., Kamei, S., and Soma, M. (2017). Clinical Study of Seven Patients with Infarction in Territories of the Anterior Inferior Cerebellar Artery. *J Stroke Cerebrovasc Dis* 26**,** 574-581.

Ogawa, Y., Otsuka, K., Hagiwara, A., Inagaki, T., Shimizu, S., Nagai, N., Konomi, U., Itani, S., Kondo, T., and Suzuki, M. (2016). Clinical evaluation of acute phase nystagmus associated with cerebellar lesions. *J Laryngol Otol* 130**,** 536-540.

Pavlin-Premrl, D., Waterston, J., Mcguigan, S., Infeld, B., Sultana, R., O'sullivan, R., and Gerraty, R.P. (2015). Importance of spontaneous nystagmus detection in the differential diagnosis of acute vertigo. *J Clin Neurosci* 22**,** 504-507.

Tarnutzer, A.A., Gold, D., Wang, Z., Robinson, K.A., Kattah, J.C., Mantokoudis, G., Saber Tehrani, A.S., Zee, D.S., Edlow, J.A., and Newman-Toker, D.E. (2023). Impact of clinician training background and stroke location on bedside diagnostic accuracy in the acute vestibular syndrome -a meta-analysis. *Ann Neurol*.

Von Brevern, M., Bertholon, P., Brandt, T., Fife, T., Imai, T., Nuti, D., and Newman-Toker, D. (2015). Benign paroxysmal positional vertigo: Diagnostic criteria. *J Vestib Res* 25**,** 105-117.

Weng, Y.C., and Young, Y.H. (2014). Mapping affected territory of anterior/posterior inferior cerebellar artery infarction using a vestibular test battery. *Acta Otolaryngol* 134**,** 268-274.

Whiting, P.F., Rutjes, A.W., Westwood, M.E., Mallett, S., Deeks, J.J., Reitsma, J.B., Leeflang, M.M., Sterne, J.A., Bossuyt, P.M., and Group, Q.-. (2011). QUADAS-2: a revised tool for the quality assessment of diagnostic accuracy studies. *Ann Intern Med* 155**,** 529-536.
